# FABP4 as a Mediator of Lipid Metabolism and Pregnant Uterine Dysfunction in Obesity

**DOI:** 10.1002/advs.202501077

**Published:** 2025-04-07

**Authors:** Xuan Li, Huihui Yu, Ruixian Tian, Xingxing Wang, Ting Xing, Chenyi Xu, Tengteng Li, Xue Du, Qianqian Cui, Biao Yu, Yunxia Cao, Zongzhi Yin

**Affiliations:** ^1^ Department of Obstetrics and Gynaecology The First Affiliated Hospital of Anhui Medical University 218 Jixi Rd Hefei Anhui Province 230022 China; ^2^ NHC Key Laboratory of the Study of Abnormal Gametes and the Reproductive Tract Anhui Medical University Hefei Anhui Province 230022 China; ^3^ Anhui Province Key Laboratory of Reproductive Disorders and Obstetrics and Gynaecology Diseases No 81 Meishan Road Hefei Anhui 230032 China; ^4^ Engineering Research Center of Biopreservation and Artificial Organs Ministry of Education No 81 Meishan Road Hefei Anhui 230032 China; ^5^ Key Laboratory of Population Health Across Life Cycle (Anhui Medical University) Ministry of Education of the People's Republic of China No 81 Meishan Road Hefei Anhui 230032 China; ^6^ Center for Big Data and Population Health of IHM Hefei Anhui 230022 China

**Keywords:** fatty acid binding protein 4 (FABP4), high‐fat diet (HFD), obesity, pregnancy, uterine contraction

## Abstract

Obese pregnant women in late pregnancy are more susceptible to uterine smooth muscle dysfunction, but the underlying mechanisms remain elusive. Here, elevated levels of fatty acid binding protein 4 (FABP4) in the myometrium of obese pregnant women at term, high‐fat diet (HFD)‐induced obese mice, and palmitic acid‐treated uterine smooth muscle cells (USMCs), are demonstrated. FABP4 plays a critical role in transporting fatty acids from the extracellular to the intracellular compartment. Mechanistically, obesity promotes excessive fatty acid uptake, leading to aberrant lipid accumulation and reduced ATP production in USMCs. These abnormalities stem from weakened coupling of mitochondria‐associated membranes, which are essential for calcium, lipids, and metabolites exchange. Furthermore, adenoviral injection to elevate FABP4 levels in normal‐diet mice mimicks the effects observed in HFD mice. Collectively, these findings highlight FABP4 as a key driver of myometrial dysfunction in obesity and a potential therapeutic target for improving labor outcomes in obese pregnancies.

## Graphical abstract

Obesity during late pregnancy contributes to uterine smooth muscle dysfunction, but the underlying mechanisms are unclear. This study identifies fatty acid binding protein 4 (FABP4) as a key player in the process, mediating excessive fatty acid uptake, lipid accumulation, and mitochondrial dysfunction in myometrial cells. FABP4 could be a novel uterine therapeutic target to improve pregnancy outcomes in obese women.

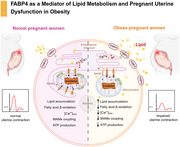

## 1 Introduction

The global obesity rate has been steadily increasing, posing a serious public health challenge and threatening sustainable human development. Similarly, obesity prevalence among women of childbearing age has risen in parallel with the general population, becoming a prevalent health concern.^[^ ^1^ ^]^ Observational studies have confirmed a link between maternal obesity and adverse pregnancy outcomes, including preeclampsia, gestational diabetes, and premature rupture of membranes. Several obesity‐related complications are associated with impaired uterine contraction during the perinatal period, with evidence strongly suggesting that obese women are at increased risk of inefficient uterine contractility during labor.^[^ ^2^, ^3^ ^]^ For instance, spontaneous contractions in myometrial strips from obese women reportedly decrease with increasing body weight,^[^ ^2^ ^]^ although these findings remain controversial.^[^ ^4^, ^5^ ^]^ Proposed mechanisms primarily focus on obesity altering hormonal and lipid levels, which in turn affect myocyte function or composition, ultimately reducing uterine contractility. However, the exact underlying mechanisms remain poorly understood.^[^ ^6^, ^7^ ^]^

Myocytes, which constitute over 95% of uterine cells, are responsible for generating electrical activity and subsequent contractions that drive labor. These myocytes exhibit minimal activity during the nonpregnant state and most of gestation, but significantly increase in excitability and contractility in the final days before parturition.^[^ ^8^, ^9^ ^]^ The intrinsic phasic activity of myocytes is tightly regulated by hormonal, metabolic, and mechanical factors.^[^ ^10^ ^]^

Besides changes in membrane potential required for initiating cell contraction, energy metabolism and substrate availability are also critical for normal contractions. Abnormal changes in metabolic substrates disrupt cellular energy production. Fatty acids are the primary metabolic fuel during delivery, followed by carbohydrates and amino acids.^[^ ^11^ ^]^ In the heart, which is composed primarily of smooth muscle, free fatty acid oxidation provides 60–90% of myocardial ATP, while glutamate and lactate provide 10–40%. Under ischemic conditions, the decline in myocardial fatty acid oxidation, leading to reduced energy production and diminished contractile capacity.^[^ ^12^ ^]^

The lipid overflow hypothesis suggests that when adipose tissue exceeds its storage capacity, excess free fatty acids spill over into other tissues, such as skeletal or cardiac muscle.^[^ ^13^ ^]^ This results in intramyocellular fat accumulation and mitochondrial stress due to incomplete fatty acid oxidation.^[^ ^14^, ^15^ ^]^ Excessive accumulation of fatty acids may lead to mitochondrial dysfunction, which in turn affects muscle contraction ability and overall metabolic status. Obesity has been shown to adversely affect skeletal muscle by disrupting the mitochondrial electron transport chain and downregulating genes involved in oxidative metabolism and mitochondrial biogenesis, such as peroxisome proliferator‐activated receptor γ (PPAR‐γ) and PPAR co‐activator 1 α (PGC‐1α).^[^ ^16^, ^17^ ^]^ Mitochondria‐associated ER membranes (MAMs) play a crucial role in lipid metabolism by facilitating the transport of fatty acids and other lipids, thus maintaining lipid homeostasis.^[^ ^18^ ^]^ Additionally, Mitofusin 2 (Mfn2), as a mitochondrial fusion protein, plays a vital role in regulating the morphology and function of mitochondria.^[^ ^19^ ^]^ The roles of MAMs and Mfn2 in maintaining mitochondrial health, promoting lipid metabolism, and supporting cellular energy demands require further investigation. Fatty acid binding protein 4 (FABP4) is a lipid‐binding protein with a highly conserved fatty acid‐binding domain, capable of efficiently binding and transporting fatty acids.^[^ ^20^ ^]^ FABP4 regulates lipid metabolism in tissues such as adipocytes, muscle, and liver, playing a crucial role, particularly in the oxidation and storage of fatty acids.^[^ ^21^, ^22^, ^23^ ^]^ However, the effects of obesity on myometrial contraction remain poorly understood, necessitating further multi‐level investigations to elucidate the underlying mechanisms.

In this study, we demonstrate that uterine contraction is impaired in obese pregnant women, high‐fat diet (HFD)‐induced obese mice, and palmitate (PA)‐treated uterine smooth muscle cells (USMCs) due to (FABP4 upregulation. FABP4 disrupts lipid metabolism by causing lipid accumulation, mitochondrial dysfunction, weakened MAMs coupling, and suppressed fatty acid utilization, primarily through carnitine palmitoyl transferase 1A (CPT1A) inhibition. Overexpression of FABP4 in normal‐diet (ND) mice confirmed its direct role in reducing uterine contraction and impairing metabolism. These findings highlight the critical role of FABP4 in obesity‐induced myometrial dysfunction, providing potential therapeutic targets for improving uterine contractility and labor outcomes in obese pregnancies.

## 2 Results

### 2.1 Weakened Uterine Contractions in Obese Myometrium

**Figure 1:**
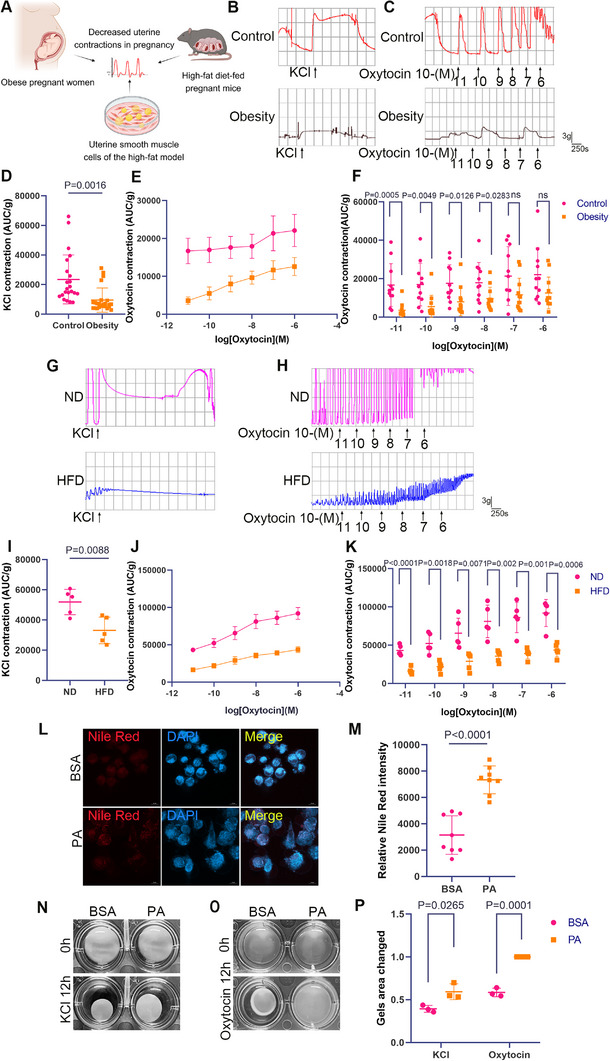
Impaired uterine contractions in the myometrium of obese individuals. A) Schematic diagram illustrating the inhibition of uterine contractions in pregnancy due to obesity and HFD, created using BioRender.com. B–F) Isometric contraction experiments were performed on uterine muscle tissues from late‐pregnancy nonlaboring normal‐weight pregnant women (control) and obese pregnant women (obesity), and the contractions were observed after KCl and oxytocin stimulation. B,D) Contraction curves and statistical results of uterine strips from the control and obesity groups under 96 mm KCl stimulation within 5 min (*n* = 22 for control group; *n* = 24 for obesity group, pooled from 3 mice per sample). C,E,F) Contraction curves and statistical results from both groups under continuous oxytocin stimulation (*n* = 11 for control group; *n* = 12 for obesity group, pooled from 3 mice per sample). G–K) Isometric contraction experiments on uterine muscle tissues from late‐pregnancy mice fed a HFD and ND mice at the same gestational age. Contractions were observed after KCl and oxytocin stimulation (*n* = 5 mice per group). G,I) Contraction curves and statistical results of uterine strips from the ND group and the HFD group under 96 mm KCl stimulation within 5 min. H,J,K) Contraction curves and statistical results of uterine strips from the ND group and the HFD group under continuous oxytocin stimulation. L–P) USMCs from late‐pregnancy mice were treated with BSA and PA, and collagen contraction experiments were performed to compare the contractile ability of the cells. The area of the floating collagen reflects the contractile ability of the cells, with a larger collagen area indicating weaker cell contractions, and vice versa. L,M) Representative photomicrographs of Nile Red staining, with the mean gray value quantified for each image (*n* = 9 in each group), scale bar = 10 µm. N–P) Collagen contraction curves and statistical results of cells under BSA and PA treatment after KCl and oxytocin stimulation, Changes in collagen contraction area were quantified (*n* = 3 mice per group). All experiments were repeated 3–4 times with consistent results. The values are expressed as the means ± SDs. ^*^ *p* < 0.05, ^**^ *p* < 0.01, and ^***^ *p* < 0.001 versus the control group.

To investigate obesity's effects on uterine contractions during late pregnancy (**Figure** **1**A), we collected uterine strips from obese or nonobese pregnancy women at term (clinical data showing Body Mass Index (BMI) differences, Figure S1A, Supporting Information) and from late‐pregnancy mice fed either ND or HFD (60% kcal from fat) for 16 weeks. The HFD group exhibited a significant increase in body weight (over 20%) and elevated plasma triglycerides (TG), cholesterol (TC), high‐density lipoprotein (HDL), and low‐density lipoprotein (LDL) levels compared to the ND group (Figure S1B,C, Supporting Information). Oil Red O staining and transmission electron microscopy (TEM) confirmed lipid accumulation in the uterine myometrium (Figure S1D,E, Supporting Information).

To investigate the effect of obesity on uterine contractions, we stimulated uterine smooth muscle strips with 96 mm potassium chloride (KCl) and graded oxytocin. The results showed that, in the first few seconds before KCl stimulation, the obese group exhibited a lower contraction frequency and significantly reduced contraction peak values. To quantitatively assess the contraction strength, we calculated the ratio of the area under the curve (AUC) to the muscle strip weight during the first 5 min, which provided the AUC/g value. The results indicated that uterine contraction strength was lower in the obese group compared to the control group (Figure 1B,D). When uterine muscle strips were stimulated with oxytocin concentrations ranging from 10^−11^ to 10^−6^ m, the results showed a concentration‐dependent increase in uterine contraction strength in both groups of pregnant women. However, the obese group exhibited overall weaker contraction ability. Within the oxytocin concentration range of 10^−11^ to 10^−8^ m, the uterine contraction ability in the obese group was significantly reduced (Figure 1C,E,F). Similarly, Isometric contraction experiments demonstrated significantly reduced uterine contractions in HFD‐fed mice compared to controls in response to KCl and graded oxytocin stimulation (Figure 1G–K).

To further investigate obesity's effects on uterine contractions, we isolated and purified primary USMCs from late‐pregnancy mice and established an immortalized late‐pregnancy USMCs cell line (Figure S1F, Supporting Information). Nile red staining revealed intramyocellular fat accumulation in PA‐treated USMCs (Figure 1L,M), accompanied by reduced fatty acid oxidation (FAO) (Figure S1G, Supporting Information). Collagen contraction assays showed a significant reduction in contractile ability in PA‐treated cells compared to the control under high‐KCl and oxytocin stimulation (Figure 1N–P). These findings collectively suggest that obesity during pregnancy, HFD‐induced obesity and PA‐induced alterations in USMCs, impair uterine contractions during labor.

### 2.2 FABP4 Protein Is Upregulated in the Myometrium of HFD‐Fed Mice

**Figure 2:**
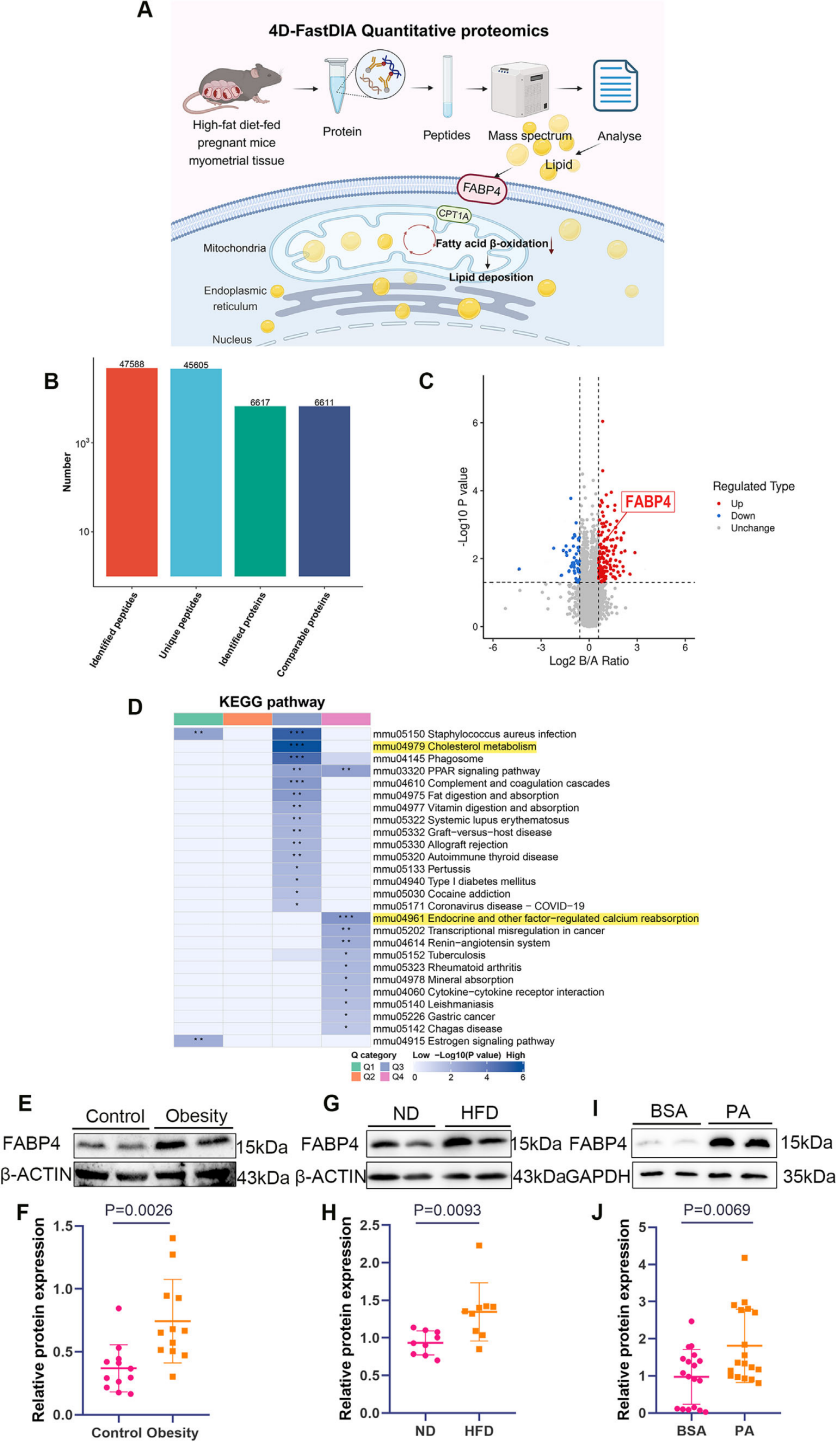
FABP4‐mediated cholesterol metabolism is enhanced in the myometrium under HFD. A) Schematic diagram of 4D‐FastDIA quantitative proteomic analysis for uterine smooth muscle tissue in the obese mouse model, created with BioRender.com. B) Basic statistical analysis of mass spectrometry data. C) Volcano plot showing the distribution of differentially expressed proteins, with red dots representing significantly upregulated proteins and blue dots representing significantly downregulated proteins. The FABP4 protein, highlighted by the red box, is a significantly elevated protein. D) KEGG enrichment and cluster analysis of Q1‐Q4. Hierarchical clustering of KEGG pathways showed that high lipid significantly activated the cholesterol metabolism pathway and impacted calcium reabsorption pathways regulated by endocrine factors. E,F) Representative Western blots and quantitative analysis of FABP4 expression in uterine smooth muscle tissue of pregnant women (*n* = 12 samples per group, pooled from 3 mice per sample). G,H) Representative Western blots and quantitative analysis of FABP4 expression in uterine smooth muscle tissue of HFD‐fed pregnant mice (*n* = 9 samples per group, pooled from 3 mice per sample). I,J) Representative Western blots and quantitative analysis of FABP4 expression in USMCs of the high‐fat model (*n* = 18 per group). All experiments were repeated 3–4 times with consistent results. The values are expressed as the means ± SDs. ^*^ *p* < 0.05, ^**^ *p* < 0.01, and ^***^ *p* < 0.001 versus the control group.

To explore obesity‐induced changes during uterine contractions, we performed 4D‐FastDIA quantitative proteomic analysis on uterine smooth muscle tissues from HFD‐fed and control mice (**Figure** **2**A). The analysis revealed alterations in the proteome of myometrium from HFD and ND mice (Figure 2B; Figure S2A, Supporting Information). Among the upregulated proteins, FABP4 emerged as a significantly increased protein and associated with lipid transport (Figure 2C; Figure S2B, Supporting Information). Gene Ontology enrichment analysis of differentially expressed proteins (DEPs) showed significant involvement in biological processes related to cholesterol transport and plasma membrane invagination (Figure S2C, Supporting Information). Cellular component analysis indicated that DEPs in the HFD group were predominantly linked to lipoprotein particles and complexes, compared to the ND group (Figure S2D, Supporting Information). Molecular function analysis indicated that the DEPs in the HFD group were primarily associated with lipoprotein binding (Figure S2E, Supporting Information).

To assess functional differences, proteins were categorized into four groups (Q1–Q4) based on their differential expression levels (Figure S2F, Supporting Information). KEGG enrichment analysis, using hierarchical clustering, highlighted the significant activation of cholesterol metabolism and endocrine‐regulated calcium reabsorption pathways in the HFD group (Figure 2D).

As a lipid chaperone that regulates lipid transport and metabolism, FABP4 was the focus of further investigation.^[^ ^20^ ^]^ Elevated FABP4 expression was validated in the uterine smooth muscle of obese pregnant women, HFD‐fed mice, and PA‐treated USMCs from late‐pregnancy mice (Figure 2E–J). Additionally, we observed a reduction in FAO in PA‐treated mouse USMCs and analyzed the expression of CPT1A, a key FAO enzyme, using Western blot and immunofluorescence. CPT1A expression was significantly reduced in both HFD‐fed mice and PA‐treated USMCs (Figure S2G–L, Supporting Information).

### 2.3 FABP4 Drives Lipid Accumulation and Impairs Uterine Contractions

**Figure 3:**
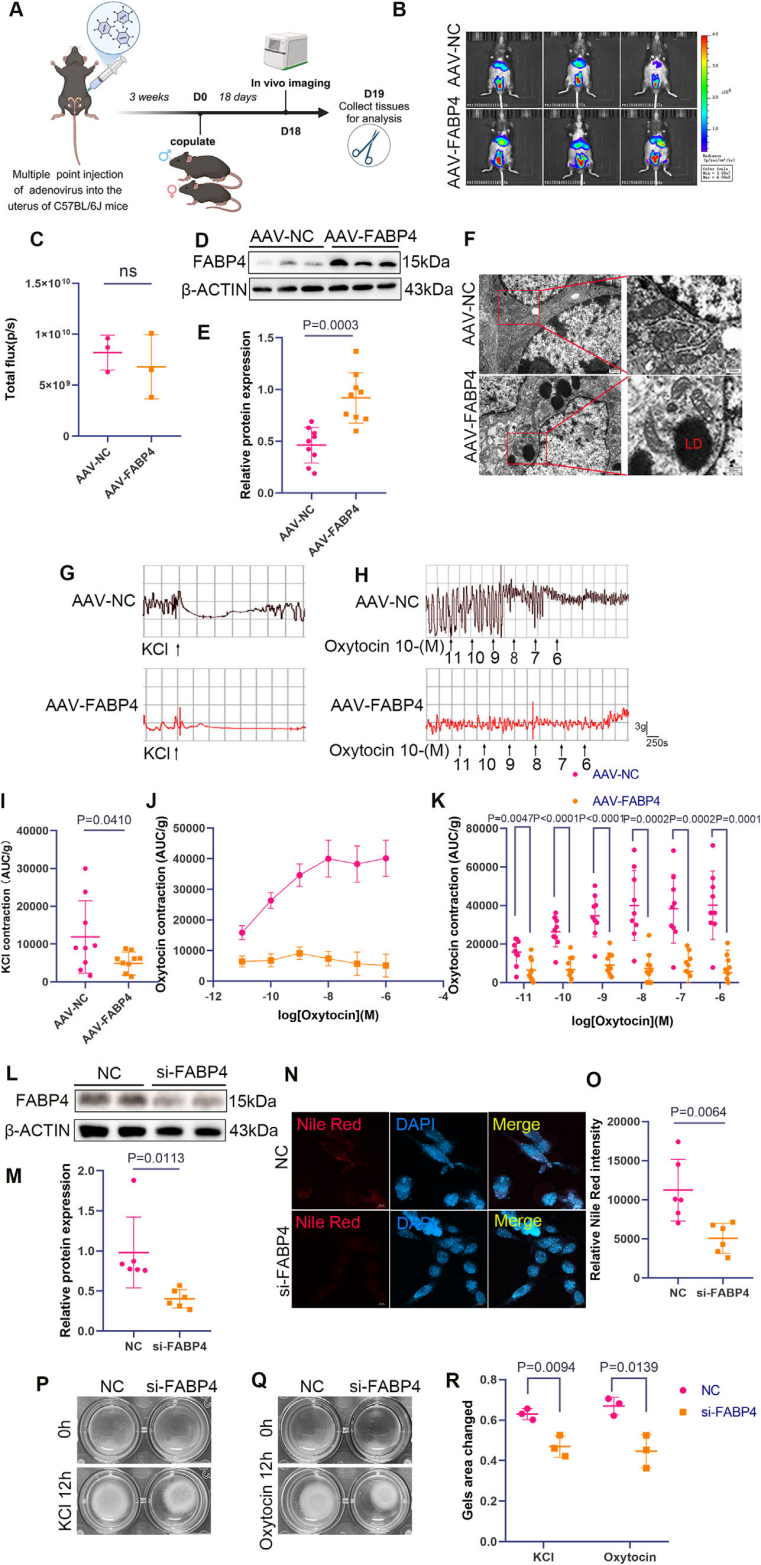
FABP4 promotes lipid accumulation and disrupts uterine contractions. A) Schematic diagram of FABP4 gene overexpression mouse model construction, created with BioRender.com. B,C) Representative images of the uterine in live mice injected with 1E+11vg of AAV2/9‐CMV‐luc, analyzed using the IVIS live imaging system (*n* = 3 per serotype). D,E) Effect of FABP4 overexpression plasmid injection (*n* = 9 samples per group, pooled from 3 mice per sample). F) Representative images showing LDs. G,I) Uterine contraction curves and statistical results under 96 mm KCl stimulation for 5 min, comparing AAV‐NC and AAV‐FABP4 groups (*n* = 9 samples per group, pooled from 3 mice per sample). H,J,K) Uterine contraction curves and statistical results under continuous oxytocin stimulation, comparing the same groups (*n* = 9 samples per group, pooled from 3 mice per sample). L,M) Western blot analysis and quantification showing FABP4 siRNA transfection efficiency (*n* = 6 samples per group, pooled from 3 mice per sample). N,O) Nile Red staining of LDs in USMCs. Quantified mean gray values for each image (*n* = 6 samples per group, pooled from 3 mice per sample), Scale bar = 10 µm. P–R) Collagen contraction images and statistical results of USMCs transfected with FABP4 siRNA and treated with PA under KCl and oxytocin stimulation (*n* = 3 mice per group). All experiments were repeated 3–4 times with consistent results. The values are expressed as the means ± SDs. ^*^ *p* < 0.05, ^**^ *p* < 0.01, and ^***^ *p* < 0.001 versus the control group.

To investigate the role of FABP4 in lipids metabolism and uterine contractions, we utilized adeno‐associated virus (AAV)‐mediated gene delivery via multiple in situ uterine injections in ND‐fed mice, a widely used method in muscle disease studies (**Figure** **3**A).^[^ ^24^, ^25^ ^]^ ND‐fed mice were intramuscularly injected with either pAAV‐CMV‐Luc2‐P2A‐3xFLAG‐FABP4‐tWPA (AAV‐FABP4 group) or pAAV‐CMV‐Luc2‐P2A‐3xFLAG‐MCS‐tWPA (AAV‐NC group) through orthotopic uterine multi‐point injections.

Luciferase activity was tracked over time using in vivo imaging, confirming enhanced successful transgene expression in the uteri of both mouse groups (Figure 3B,C). FABP4 protein expression and localization were confirmed by immunoblotting and immunohistochemistry (Figure 3D,E; Figure S3A,B, Supporting Information). TEM revealed cytoplasmic lipid droplets (LDs) in USMCs of the AAV‐FABP4 group (Figure 3F). Isometric contraction experiments on myofibrils demonstrated that FABP4 overexpression reduces the contractile ability of the uterine myometrium (Figure 3G–K). Notably, FABP4 overexpression led to a decrease in CPT1A levels (Figure S3C,D, Supporting Information), suggesting that FABP4 regulates lipid metabolism through CPT1A and FABP4 overexpression induces lipid accumulation, mitochondrial dysfunction, and impaired uterine contraction.

Next, we examined the effects of FABP4 silencing on lipids metabolism and uterine contractions. Using siRNA‐mediated FABP4 knockdown (KD) in PA‐treated mouse USMCs from late pregnancy, we confirmed the KD efficiency (Figure 3L,M; Figure S3E, Supporting Information) and observed reduced lipid accumulation in PA‐treated cells, as shown by Nile Red staining (Figure 3N,O). Consistent with isotonic myofibril contraction results, collagen contraction assays demonstrated that FABP4 silencing enhanced contraction in PA‐treated USMCs under high‐KCl and oxytocin stimulation (Figure 3P–R). These findings further confirm that FABP4 regulates lipid metabolism and uterine contraction. FABP4 promotes fatty acid accumulation by inhibiting FAO via CPT1A downregulation. We then overexpressed CPT1A via plasmid transfection (Figure S3F,G, Supporting Information) and observed enhanced uterine contraction after incubating the USMCs with PA (Figure S3H–K), Supporting Information, indicating that FABP4 regulates lipid metabolism through CPT1A.

Beyond regulating lipid metabolism, proteomic results revealed differences in glucose metabolism between groups. Studies have shown that fatty acids and carbohydrates are the main metabolic fuel during delivery.^[^ ^11^, ^12^ ^]^ The proteomics results also showed that differentially expressed proteins are present in the three major metabolic pathways in the COG/KOG functional classification distribution (Figure S4A, Supporting Information). We examined key proteins involved in glucose metabolism and found that hypoxia‐inducible factor‐1 alpha (HIF‐1α), glucose transporter 1 and 4 (GLUT1, GLUT4), pyruvate dehydrogenase kinase 1 (PDK1), and lactate dehydrogenase A (LDHA) were significantly downregulated (Figure S4B,C, Supporting Information). Consistent with these findings, lactate levels were increased in the myometrium of HFD‐fed mice and PA‐treated USMCs (Figure S4D,E, Supporting Information). Moreover, CPT1A levels were reduced following lactate treatment, suggesting that glycolysis regulates FAO via CPT1A (Figure S4F–H, Supporting Information).

To explore how HFD influences glucose metabolism and insulin signaling in the myometrium of pregnant mice, we evaluated protein kinase B (PKB/AKT) phosphorylation and measured glucose uptake, which quantifies cellular uptake of 2‐deoxyglucose‐6‐phosphate (2DG6P). The PA group exhibited significantly reduced AKT phosphorylation upon insulin stimulation (Figure S4I, Supporting Information) and diminished glucose uptake compared to the control group (Figure S4K, Supporting Information), indicating insulin resistance and impaired glucose uptake due to HFD.

### 2.4 Mitochondrial Dysfunction Underlies Weakened Uterine Contractions

**Figure 4:**
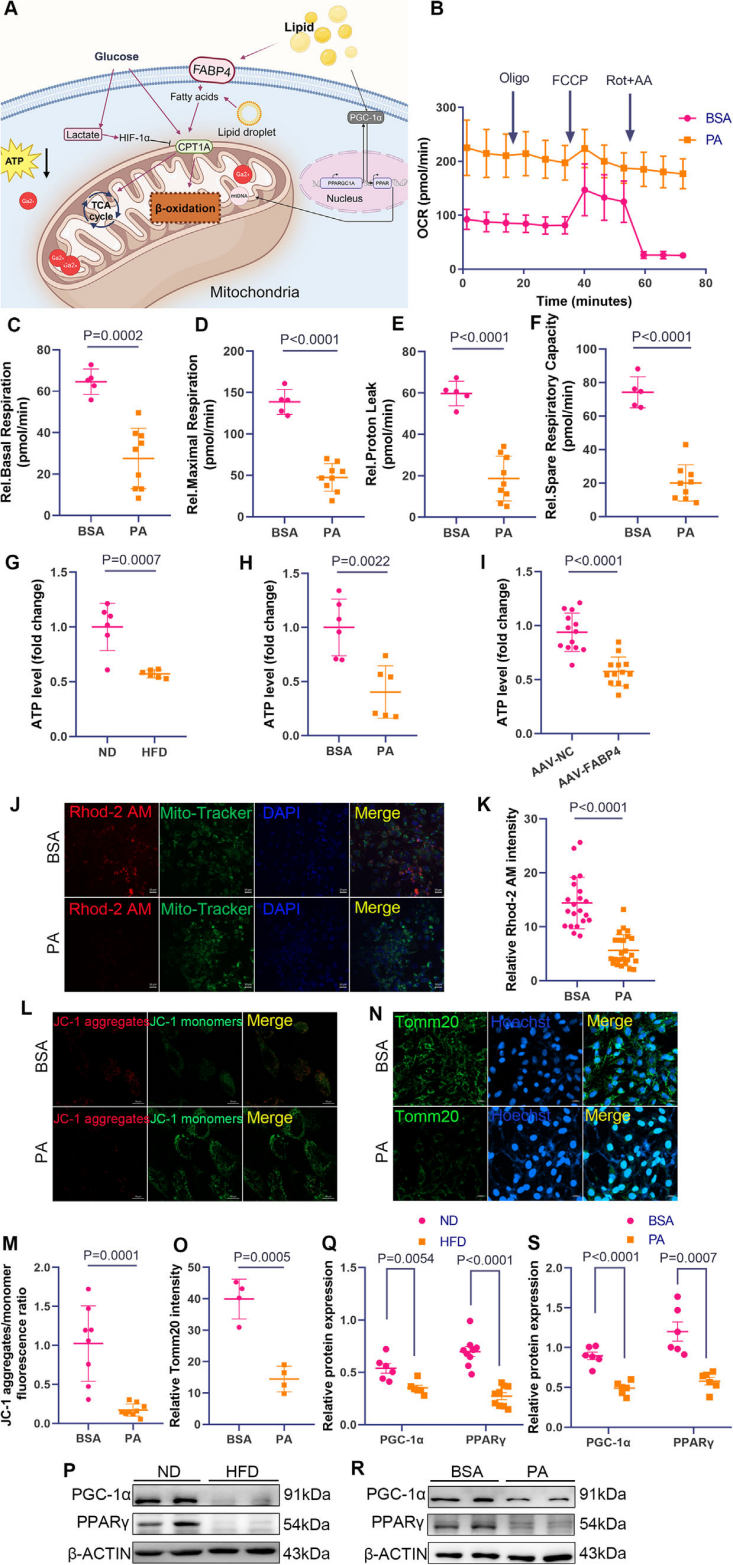
Mitochondrial dysfunction contributes to weakened uterine contractions. A) Schematic diagram of mitochondrial dysfunction caused by obesity and HFD, created with BioRender.com. B–F) Representative line and bar graphs of OCR in PA‐induced USMCs, including basal respiration, maximal respiration, respiratory reserve capacity, proton leak, and ATP production (*n* = 5 for BSA group, *n* = 9 for PA group; samples pooled from 3 mice per sample). G) The ATP levels in the uterine myometrium of late‐pregnant obese mice (*n* = 6 samples per group, pooled from 3 mice per sample). H) The ATP levels in the PA‐treated cells (*n* = 6 per group). I) The ATP levels in the uterine myometrium of late‐pregnant mice with AAV injection, measured using the ATP Assay Kit (*n* = 13 for AAV‐NC group, *n* = 16 for AAV‐FABP4 group; pooled from 3 mice per sample). J,K) Representative images of Rhod‐2 AM (red) staining for mitochondrial calcium and Mito‐Tracker (green) labeling for mitochondria in USMCs (*n* = 21 for BSA group, *n* = 24 for PA group; pooled from 3 mice per sample), scale bar = 20 µm. L,M) Mitochondrial membrane potential analysis using the JC‐1 probe (*n* = 8 in the BSA group and *n* = 9 in the PA group), scale bar = 20 µm. N,O) Immunofluorescent images of uterine myometrium stained with TOMM20 (green) and nuclei (blue) in BSA‐ and PA‐treated groups (*n* = 4 per group), scale bar = 10 µm. P,Q) Western blot and quantitative analysis of PGC‐1α and PPAR γ expression in uterine smooth muscle tissue from HFD pregnant mice (*n* = 6 for control group, *n* = 9 for obesity group; pooled from 3 mice per sample). R,S) Western blot and quantitative analysis of PGC‐1α and PPAR γ expression in USMCs treated with BSA or PA (*n* = 6 per group). All experiments were repeated 2–3 times with consistent results. The values are expressed as the means ± SDs. ^*^ *p* < 0.05, ^**^ *p* < 0.01, and ^***^ *p* < 0.001 versus the control group.

Mitochondrial swelling observed in the AAV‐FABP4 group suggests that mitochondria play a role in the FABP4‐induced weakening of uterine contractions. To evaluate mitochondrial function, we assessed mitochondrial oxygen consumption rate (OCR) in PA‐treated cells to evaluate mitochondrial function (**Figure** **4**A). PA treatment reduced mitochondrial OCR in USMCs (Figure 4B–F). Since mitochondrial β‐oxidation of fatty acids generates ATP, we measured ATP production and found that it was reduced in USMCs from HFD‐fed mice compared to ND‐fed controls (Figure 4G). Similarly, ATP production was decreased in PA‐treated USMCs (Figure 4H). Notably, in AAV‐FABP4 mice fed an ND, ATP production was also reduced, indicating FABP4 directly impairs energy metabolism, independent of obesity or HFD (Figure 4I).

Reduced mitochondrial calcium disrupts membrane potential and oxidative respiration. Rhod‐2 AM staining revealed decreased mitochondrial calcium in PA‐treated cells (Figure 4J,K). JC‐1 staining showed lipid‐induced impairment of mitochondrial membrane potential (Figure 4L,M).

TOMM20 staining further showed a disrupted mitochondrial network in PA‐treated USMCs (Figure 4N,O). Western blotting revealed downregulation of PPAR‐γ and PGC‐1α, key regulators of mitochondrial biogenesis, in both HFD myometrium and PA‐treated USMCs (Figure 4P–S). Immunofluorescence confirmed these findings, showing reduced PPAR‐γ and PGC‐1α expression in PA‐treated USMCs (Figure S5A–D, Supporting Information). Together, these results suggest the weakened uterine contractions of USMCs caused by PA treatment is associated with mitochondrial disruption.

### 2.5 PA‐Induced MAMs Disruption Inhibits Uterine Contractions

DEPs identified by 4D‐FastDIA proteomics were localized in various cellular compartments, including the extracellular space, nucleus, cytoplasm, mitochondria, plasma membrane, and endoplasmic reticulum (ER) (Figure S5E, Supporting Information). MAMs play key roles in steroid synthesis, phospholipid metabolism, and mitochondrial bioenergetics.^[^ ^18^ ^]^ In both AAV‐FABP4 mice and PA treated USMCs, we observed wider distances between the ER and mitochondria compared to controls, suggesting that obesity or PA treatment impairs MAMs and related proteins (Figure 3F; Figure S1I, Supporting Information).

**Figure 5:**
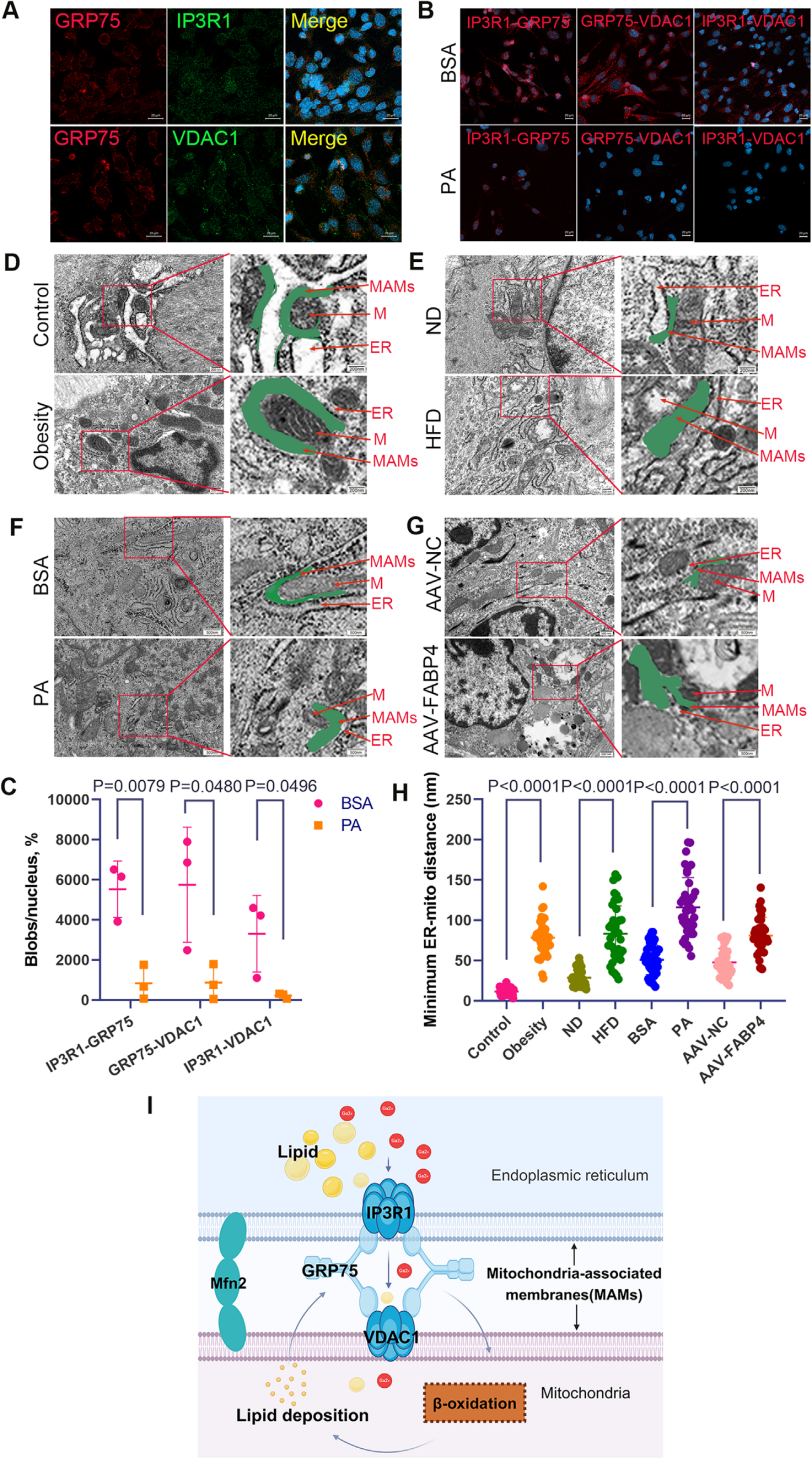
PA‐induced disruption of MAMs impairs uterine contractions. A) Immunofluorescence analysis showing the co‐localization of IP3R1, GRP75, and VDAC1 in USMCs (*n* = 3 mice per group), scale bar = 20µm. B,C) PLA detects changes in ER‐mitochondria interactions related to the IP3R1‐GRP75‐VDAC1 complex. Representative PLA images and quantitative analysis of the interactions between IP3R1‐GRP75, GRP75‐VDAC1, and IP3R1‐VDAC1 in USMCs treated with BSA or PA for 24 h (*n* = 3 mice per group), scale bar = 20µm. D–H) Representative electron microscopy images showing mitochondria (M), ER, and MAMs (arrows). Calculations of the shortest distances between ER and mitochondrial membranes were made (*n* > 40 ER‐mitochondria contact points per group; pooled from 3 mice per sample), image scale = 200 nm or 500nm. I, Schematic representation of obesity and HFD‐induced damage to MAMs, created with BioRender.com. All experiments were repeated 3 times with consistent results. The values are expressed as the means ± SDs. ^*^ *p* < 0.05, ^**^ *p* < 0.01, and ^***^ *p* < 0.001 versus the control group. Two‐tailed Student's *t*‐test was used for PLA fluorescence signal analysis.

The inositol 1, 4, 5‐trisphosphate receptor 1‐glucose‐regulated protein 75‐voltage‐dependent anion channel 1(IP3R1‐GRP75‐VDAC1) complex directly controls calcium transfer from the ER to the mitochondria.^[^ ^26^ ^]^ Immunofluorescence confirmed the co‐localization of IP3R1, GRP75, and VDAC1 in USMCs, supporting their role in ER‐mitochondria interactions (**Figure** **5**A). In addition, in situ proximity ligation assay (PLA) quantified interactions among IP3R1, GRP75, and VDAC1. Using a Texas Red‐labeled oligonucleotide probe, we visualized ER‐mitochondria tethering, where each fluorescent dot represents an IP3R1‐VDAC1 interaction. In PA‐treated USMCs, fluorescence signals for IP3R1‐VDAC1, IP3R1‐GRP75, and GRP75‐VDAC1 interactions were reduced (Figure 5B,C).

To assess the impact of obesity and HFD affect energy production, lipid metabolism, and uterine contraction through MAMs disruption, we performed TEM, which showed increased distances between the ER and mitochondrial membranes in obesity, HFD‐fed mice, PA‐treated USMCs, and AAV‐FABP4 group indicating disrupted MAMs. These results were confirmed by significantly increased ER‐mitochondria distances in all four experimental conditions (Figure 5D–H). Together, these findings suggest that PA treatment impairs uterine contraction by reducing MAMs coupling (Figure 5I).

### 2.6 Mfn2 Upregulation Restores MAMs Coupling and Improves Uterine Contractions

**Figure 6:**
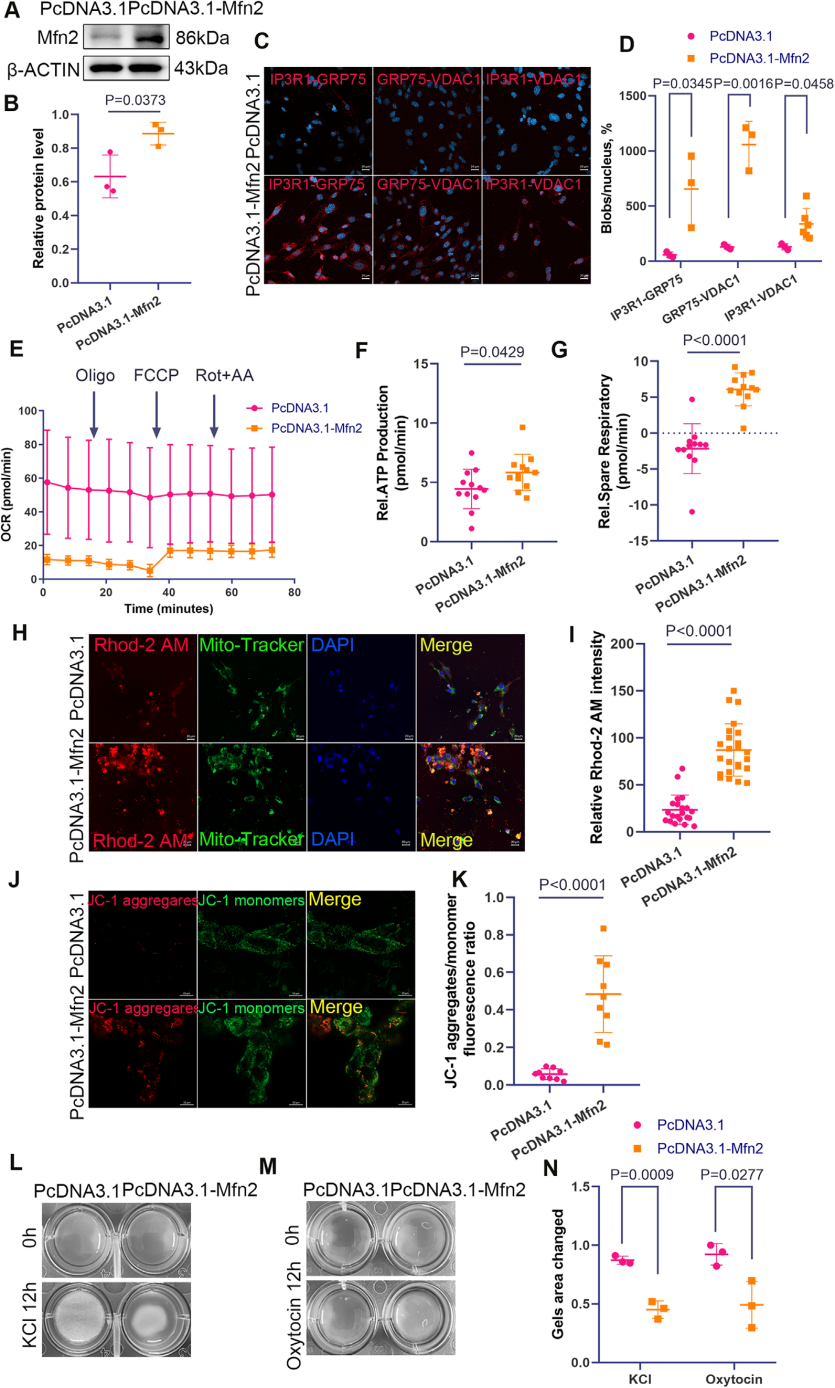
Upregulation of Mfn2 restores MAMs coupling and enhances uterine contractions. A,B) Effect of Mfn2 overexpression plasmid transfection for 48 h (*n* = 3 mice per group). C,D) Representative immunoprecipitation images showing the interaction between VDAC1, GRP75, and IP3R1 in USMCs transfected with Mfn2 overexpression plasmid and treated with BSA or PA (200 µm) for 24 h (*n* = 3 mice per group), scale bar = 20 µm. E–G) Representative line and bar graphs of OCR measurements in PA‐induced USMCs, including basal respiration, maximal respiration, ATP production, and respiratory reserve capacity (*n* = 12 for each group). H,I) Representative images of Rhod‐2 AM (red) staining for mitochondrial calcium and MitoTracker (green) labeling for mitochondria in USMCs (*n* = 21 for PcDNA3.1 group, *n* = 24 for PcDNA3.1‐Mfn2 group), scale bar = 20 µm. J,K) Mitochondrial membrane potential analysis using JC‐1 probe in USMCs. (*n* = 4 for PcDNA3.1 group, *n* = 9 for PcDNA3.1‐Mfn2 group), scale bar = 20 µm. L–N) Gel contraction images and statistical results of USMCs under conditions of Mfn2 overexpression plasmid transfection and PA treatment, with KCl and oxytocin stimulation (*n* = 3 mice per group). All experiments were repeated 2–3 times with consistent results. The values are expressed as the means ± SDs. ^*^ *p* < 0.05, ^**^ *p* < 0.01, and ^***^ *p* < 0.001 versus the control group. Two‐tailed Student's *t*‐test was used for PLA fluorescence signal analysis.

Mfn2, as a tethering protein that connects the ER and mitochondria, promotes MAMs coupling and rescues mitochondrial function when upregulated.^[^ ^27^ ^]^ Here, we investigate whether Mfn2 overexpression could restore uterine contractions and mitochondrial function in PA‐treated cells. Western blot analysis showed an increase in Mfn2 expression (**Figure** **6**A,B). As expected, Mfn2 overexpression enhanced VDAC1, GRP75, and IP3R1 interactions in PA treated USMCs (Figure 6C,D). Interestingly, increasing organelle coupling through Mfn2 overexpression restored insulin signaling and glucose uptake in PA‐treated USMCs (Figure S5F–H, Supporting Information). OCR showed that Mfn2 overexpression improved ATP production and spare respiratory capacity in PA‐treated USMCs (Figure 6E–G). Additionally, Rhod‐2 AM staining confirmed an increase in mitochondrial calcium following Mfn2 overexpression, while JC‐1 staining showed restoration of mitochondrial membrane potential (Figure 6H–K).

To further elucidate the role of MAMs in regulating uterine contraction, Mfn2 overexpression enhanced uterine contraction in PA‐treated USMCs, as shown by reduced gel area after KCl or oxytocin stimulation (Figure 6L–N). In summary, enhancing ER‐mitochondria connection via Mfn2 restores mitochondrial function and improves uterine contractions in USMCs.

## 3 Discussion

We investigated the impact of obesity on uterine contractions during late pregnancy and identified FABP4‐mediated cholesterol metabolism as a key pathway in vivo and in vitro levels. FABP4 overexpression induced lipid accumulation, mitochondrial dysfunction, and weakened uterine contraction by disrupting MAMs integrity, reducing fatty acid utilization, and ATP production. These findings reveal FABP4 as a crucial regulator of obesity‐related myometrial dysfunction, offering a potential target for therapeutic interventions to improve labor outcomes in obese pregnancies.

Myometrium contractions occur throughout pregnancy and are involved in the initiation of labor.^[^ ^28^ ^]^ The initiation of myometrium contraction occurs when the resting membrane potential of the cell transitions to an action potential in response to excitatory signals, leading to intracellular and extracellular ion flow. Our previous studies demonstrated that the high expression of the two‐pore potassium channel protein (TWIK‐related potassium channel 1) on the membrane of pregnant USMCs is critical for maintaining the resting membrane potential of myometrium and generating action potentials.^[^ ^29^ ^]^ Here, we present a novel mechanism through which uterine contractions are regulated by metabolites.

FABPs are a class of lipid chaperones that reversibly bind hydrophobic ligands, including long‐chain fatty acids and oleic acid, thereby regulating lipid circulation, transport, and metabolism.^[^ ^20^ ^]^ FABP4, known as A‐FABP or aP2, is a key member of the FABP family. Recent studies have reported that exogenous FABP4 inhibits myocardial cell contraction.^[^ ^29^ ^]^ Increased plasma FABP4 levels are positively correlated with BMI and insulin resistance index, and are also associated with lipid metabolism disorders.^[^ ^30^ ^]^ Increased FABP4 expression was observed in the myometrium of obese pregnant women, the myometrium of HFD mice, and the PA‐treated USMCs. Silencing FABP4 with siRNA restored impaired myometrium contractions.

To determine whether elevated FABP4 directly inhibits uterine contractions independent of obesity, AAV vector intramural multipoint injections were used to increase FABP4 expression in ND‐fed mice. Significant lipid accumulation in the uterine smooth muscle, with lipids stored as droplets within cells, inhibited myometrial contractions, indicating that elevated FABP4 directly impairs uterine contractions independent of obesity or HFD. Effective gene therapy strategies to regulating uterine contractions remain elusive. We report an optimized uterine‐targeted gene delivery strategy using AAV administered via uterine orthotopic multi‐point injection in mice.

Several studies suggested that LDs are tethered to mitochondria for FAO,^[^ ^31^, ^32^ ^]^ we found that CPT1A expression was reduced in the PA‐treated USMCs from late‐pregnancy mice, resulting in blocked mitochondrial FAO. Plasmid transfection to overexpress CPT1A rescued the inhibited myometrial contractions in PA‐treated USMCs. Elevated FABP4 expression facilitates increased lipid entry into cells but simultaneously downregulates CPT1A under PA treatment. This impairment in lipid metabolism results in lipid accumulation within the cells.

Proteomic analysis revealed decreased glucose metabolism in the HFD group. Lactate is produced under normoxic conditions in the myometrium and transported out of the myocyte by a family of proton‐linked monocarboxylate transporters.^[^ ^33^ ^]^ Lactate efflux increases significantly under hypoxic conditions.^[^ ^34^ ^]^ Elevated lactate levels suppress CPT1A expression, suggesting that lactate accumulation reduces fatty acid β‐oxidation and promotes lipid accumulation within cells.

Obesity increases the risk of weak myometrial contractions during delivery. Reduced mitochondrial capacity or quantity, leading to insufficient energy supply, may underlie the decreased efficiency of uterine contractions during labor. Notably, our results show that PA treatment damages mitochondrial respiratory function in USMCs, reduces mitochondrial calcium influx, and impairs mitochondrial membrane potential. Mitochondrial Ca^2+^ signaling is crucial for skeletal muscle contraction and relaxation, as it must strategically coordinate ATP production with the metabolic demands of action potential generation.^[^ ^35^ ^]^ Reduced mitochondrial Ca^2+^ uptake has been reported in diabetic cardiomyopathy.^[^ ^36^ ^]^ Our study found that PA‐treated USMCs exhibit decreased mitochondrial Ca^2+^ influx and reduced mitochondrial membrane potential, indicating impaired mitochondrial function.

Mitochondrial biogenesis is the main component of mitochondrial mass regulation and may partially mitigate mitochondrial protein depletion, maintaining functionality and energy status in critically ill patients.^[^ ^37^ ^]^ Mitochondrial synthesis is regulated by PGC‐1α and PPAR γ. Acetylcholine promoted mitochondria biogenesis via the PGC‐1α pathway and improved mitochondrial function.^[^ ^38^ ^]^ PPAR γ primarily regulates human dendritic cell function by modulating lipid metabolism.^[^ ^39^ ^]^ We observed decreased PGC‐1α and PPAR γ levels in PA‐treated USMCs, indicating that PA treatment reduces mitochondrial ATP production and biogenesis.

We subsequently investigated the mechanisms underlying mitochondrial damage caused by PA treatment. Mitochondrial function relies on the maintenance of normal lipid composition, which depends on mitochondrial phospholipid synthesis and lipid trafficking from the ER.^[^ ^40^ ^]^ Specifically, phosphatidylserine is primarily synthesized in the ER and imported into mitochondria through transient membrane contacts between MAMs and the mitochondrial outer membrane.^[^ ^41^ ^]^ The regulation of MAMs formation or signaling pathways at the MAMs surface modulates mitochondrial function, calcium influx, membrane potential, and lipid metabolism.^[^ ^42^, ^43^ ^]^ Studies show that classical MAMs proteins, including IP3R, GRP75, and VDAC1, form the IP3R‐GRP75‐VDAC1 complex, the main structure for Ca^2+^ transport from the ER to mitochondria.^[^ ^44^, ^45^ ^]^ However, the role of MAMs in uterine smooth muscle remains poorly understood. Our study found that HFD or PA treatment increases the ER‐mitochondria distance in USMCs, weakens the IP3R1‐GRP75‐VDAC1 complex interaction, and reduces calcium flux from the ER to mitochondria potentially inhibiting myometrial contractions during pregnancy. Additionally, we utilized an in situ lactate polymerization and immunocell fluorescence‐based method to detect and quantify ER‐mitochondria interactions, revealing a new role of the MAMs interface in regulating electrical and structural remodeling.

Mfn2 is a mitochondrial membrane protein that connects ER membranes to mitochondria, and its depletion causes ER stress and disrupts mitochondrial metabolism, insulin signaling, and energy homeostasis.^[^ ^19^, ^46^ ^]^ Hernández‐Alvarez, María Isabel et al. showed a reduction in Mfn2 expression in liver tissues of patients with nonalcoholic fatty liver disease and in mouse models.^[^ ^47^ ^]^ Astrocyte‐specific conditional deletion of Mfn2 suppressed perivascular mitochondrial clustering and disrupted mitochondria‐ ER contact sites.^[^ ^48^ ^]^ Our study suggest that PA treatment impairs lipid transport in MAMs, leading to lipid accumulation in USMCs, decreased lipids available for β‐oxidation, reduced ATP production, and impaired uterine contractions. Recent studies indicate that MAMs integrity is crucial for insulin action in the liver and muscles and is disrupted in hepatic and muscular insulin resistance.^[^ ^49^, ^50^ ^]^ Targeting MAMs may provide a novel strategy to improve insulin sensitivity and restore glucose homeostasis. Our study find that alterations in MAMs function under insulin‐resistant conditions, as evidenced by a reduced transfer of Ca^2+^ from the ER to mitochondria in PA‐treated uterine smooth muscle, suggesting that the regulation of Ca^2+^ signaling and exchange between organelles may contribute to muscular insulin resistance. Mfn2 overexpression targeting MAMs prevents PA‐induced alterations in insulin signaling. Taken together, we found that lipids enter the cells via FABP4 and are regulated by MAMs to modulate lipid metabolism. Upregulating the MAMs tethering protein Mfn2 restores FABP4‐mediated mitochondrial damage and enhances uterine contractions.

There are several limitations that warrant further investigation. First, the use of animal models and in vitro cell cultures, although informative, may not fully recapitulate the complexity of human obesity and pregnancy. Human‐specific factors, which involve hormonal fluctuations during pregnancy and genetic variability, may influence the observed mechanisms in ways not captured by these models. Additionally, while we have identified key molecular pathways involving FABP4 and MAMs, the long‐term effects of targeting these pathways on pregnancy outcomes and maternal health remain unclear. Future studies should aim to validate these findings in human tissues and explore the therapeutic potential of targeting FABP4 and MAMs in clinical settings. Moreover, considering the technical challenges associated with using obese pregnant mice for FABP4 gene knockout and knockdown, the potential for compensatory mechanisms in response to FABP4 inhibition has not been fully addressed, which could influence the efficacy of such interventions. Lastly, our study primarily focuses on metabolic disruptions, but additional factors such as inflammation, oxidative stress, and extracellular matrix remodeling could also contribute to myometrial dysfunction in obesity, warranting a more comprehensive approach to understanding the full spectrum of obesity‐related pregnancy complications.

## 4 Conclusion

In conclusion, our study reveals that disrupted organelle communication contributes to obesity‐induced insulin resistance, mitochondrial dysfunction, and reduced uterine contractions. Using 4D‐FastDIA proteomics, we identified FABP4‐mediated cholesterol metabolism as a key pathway upregulated in HFD‐fed mice. Elevated FABP4 levels drive lipid accumulation, weaken MAMs coupling, and impair uterine contractions, independent of obesity. FABP4 KD restored contractility, while Mfn2 overexpression improved mitochondrial function and alleviated insulin resistance. These findings uncover mechanisms by which obesity disrupts myometrial energy metabolism and highlight FABP4 and MAMs as potential therapeutic targets to improve labor outcomes in obese pregnancies.

## 5 Experimental Section

### Human Tissue

Six pregnant adult women with a singleton pregnancy attending antenatal care at the First Affiliated Hospital of Anhui Medical University, Hefei and having a term elective Caesarean section were included in a random sequence during the period September 2023 to January 2024. Three were normal weight (BMI 22.4‐23.9 kg m^−2^) and three were obese (BMI≥30 kg m^−2^) (WHO Expert Committee on Physical Status, 1995). The exclusion criteria were a known substance addiction including smoking, known comorbidities including diabetes or psychiatric illnesses or the use of medicine known to affect muscular contractions. Age, gestational age, parity and indication for Caesarean section were obtained from the medical records. Myometrial biopsies (length 2 cm × depth 0.5 cm × height 1 cm, approximately 2–3 g) were isolated from the upper part of the uterine incision (transverse isthmic incision). The main part of the biopsies was immediately soaked in ice‐cold PBS solution for Uterine Contraction Measurement. The remaining parts were either frozen in liquid nitrogen (≈200–250 mg of myometrium) and placed in a −80 °C freezer for subsequent biochemical analyses, or immersion‐fixed in 2% paraformaldehyde and 0.1% glutaraldehyde (≈8 mm^3^) for subsequent histology analyses.

### Uterine Contraction Measurement

As previously described,^[^ ^51^ ^]^ fresh muscle strips with excess connective tissue removed were longitudinally trimmed along the axis of the muscle fibers to generate endometrial strips measuring 3 mm in width and 7 mm in length. One end of each muscle strip was attached to a fixed metal hook, while the other end was connected to a tension sensor (Chengdu Instrument Factory, China) via an adjustable metal hook. The muscle strips were suspended in a 37 °C organ bath containing 10 mL of Krebs solution and continuously infused with appropriate 95% O_2_ and 5% CO_2_ in a water bath. The muscle strips were subjected to a basal tension of 1.5 g, and the contraction curve was recorded. Once a smooth and regular contraction curve appeared, the muscle strips were stimulated with 96 mm KCl and progressively increasing concentrations of oxytocin. After the experiment, the muscle strips were weighed, and the data were processed using installed data processing software to calculate the AUC. The AUC was divided by the weight of the muscle strip to obtain AUC/g for quantitative analysis.

### Animals—Establishment of an Obesity Mouse Late Pregnancy Animal Model

Female C57BL/6J mice were selected as they have reproductive organs including ovaries, fallopian tubes, uterus, and vagina, and their estrous cycle is relatively fixed, similar to humans. The mice were divided into two groups: ND group and HFD group, with 5 mice in each group, to ensure an adequate number of primary USMCs or uterine muscle strips for further experiments. After 14 weeks of feeding, mice in the HFD group with a body weight equal to or greater than 20% of the mean body weight of mice in the ND group were considered successful models. Female mice were housed with fertile male mice of the same strain fed a regular diet. The presence of a vaginal plug in female mice was checked the next morning, and the day of plug detection was considered day 0 of pregnancy (D0). On day 19 of pregnancy (D19), mice were euthanized using CO_2_ asphyxiation, and the uterus was dissected to extract primary USMCs or prepare uterine muscle strips for further experiments. Tissue specimens were stored for subsequent studies.

### Animals—Blood Biochemical Index Detection

The plasma levels of TC, TG, HDL‐c, and LDL‐c in mice were measured using the TC Assay Kit (Cat# ADS‐W‐ZF014), TG Assay Kit (Cat# ADS‐W‐ZF013), HDL‐c Assay Kit (Cat# ADS‐F‐D011), and LDL‐c Assay Kit (Cat# ADS‐F‐D012) respectively.

### Animals—Oil red O Staining

The isolated uterus of mice was fixed with 4% paraformaldehyde at low temperatures, and dehydrated with 30% sucrose. Then the tissue was embedded in optimal cutting temperature (OCT) compound, cut into 4‐µm sections, and then stained with oil red O dye for 30 min. After washed with PBS, the nucleus was re‐stained with hematoxylin and photographed.

### Animals—FABP4 Overexpression Mice by AAV

Twelve‐week‐old female C57BL/6J mice (15–20 g) were obtained from Beijing Vital River Laboratory Animal Technology Co., Ltd. All animal studies were conducted in accordance with the Anhui Medical University. Mice were housed in a controlled temperature range (72–74 F) on a 12‐h light, 12‐h dark cycle, and they were given food and water ad libitum. The mice were divided into two groups randomly (*n* = 3). Group 2 were injected with AAV vectors (pAAV‐CMV‐Luc2‐P2A‐3xFLAG‐FABP4‐tWPA) at a dose of 1E+11vg via uterine orthotopic multi‐point. Group 1 received an injection of negative control AAV vectors (pAAV‐CMV‐Luc2‐P2A‐3xFLAG‐MCS‐tWPA) at the same dose. The vectors were obtained from Heyuan Biotechnology (Shanghai) Co., Ltd. The Hamilton 800 series microliter in yeetor (100 µL) was used to inject the virus solution. Then 80‐µL AAV virus (the concentration of the diluted AAV virus was 1 × 10^10 vg mL^−1^, the dose was 8 × 10^8 vg) was injected at four different points between the uterine muscle walls with 20 µL per point. At 3 weeks post‐injection, male and female mice were caged for mating, the vaginal opening and bedding of the female mice were examined the next morning, and the mice with vaginal plugs observed were considered as D0, and we visualized luminescence in live mice and explanted tissues using the IVIS imaging system in vivo on the D18, on D19, mice were euthanized using CO_2_ asphyxiation, and the uterus was dissected to extract primary USMCs or prepare uterine muscle strips for further experiments. Tissue specimens were stored for subsequent studies.

### Animals—Luciferase Imaging in Live Animals and Explanted Tissues

Mice injected with AAV‐luc were analyzed using the IVIS Imaging System 200 Series (Caliper Life Sciences, Hopkinton, MA, USA) 10 min after intraperitoneal injection of luciferin (Caliper, Hopkinton, MA, USA) as previously described, and scanned with the IVIS (Caliper Life Sciences). Quantitative signal analysis was performed using the Living Image 2.5 software (Caliper Life Sciences). Luminescence was expressed in Total flux (photons per sec).

### TEM Detection

Tissues and cells from each group were fixed with 1 mL of glutaraldehyde and kept at 4 °C. After washing the cells with PBS twice, a certain amount of trypsin cell digestion solution was added and incubated at room temperature. When the cells became round after observation under a light microscope, a certain amount of high‐glucose DMEM medium containing 10% serum was added to terminate the digestion. After gently pipetting 3–5 times, the cell suspension was centrifuged at 1000 rpm for 5 min, and the cell pellet was gently resuspended in PBS. Glutaraldehyde was gently added to the cell pellet and kept at 4 °C for fixation. The tissue and cell samples were then fixed with osmium tetroxide, dehydrated with ethanol, rinsed with propylene oxide, embedded, and sectioned. TEM was used to obtain images of cellular structures at a magnification of 50 000x.

### Cells—Primary Culture of Late Pregnant Mouse USMCs and Cell Immortalization

Approximately 1 g of tissue was cut into 1 mm × 1 mm × 1 mm pieces and digested with 0.4% collagenase II at 37 °C in a 95% CO_2_ incubator for 4 h. The digested suspension was filtered through a 100 µm filter, and the filtrate was collected. After centrifugation at 1000 g for 5 min, the supernatant was removed, and the pellet of cells was resuspended in 5 mL of complete culture medium. The cell suspension was seeded in culture dishes and cultured until the cells reached 3/4 confluence.

Primary mouse USMCs are washed three times with PBS, then digested with 0.25% trypsin‐EDTA in a 37 °C incubator for 5 min. The digestion is terminated with a complete medium (high‐glucose DMEM + 10% FBS + 1% penicillin/streptomycin), and the cell pellet is resuspended in a complete medium and plated onto a 6 cm culture dish. When the cells reach 50% confluence, they are infected overnight with lentivirus (pGMLV‐SV40T‐PURO) at an MOI of 80, with 5 µg mL^−1^ Polybrene to enhance the efficiency of lentiviral infection. The cells are incubated at 37 °C, 5% CO_2_. The medium is changed the following day, and the cells are continuously cultured for another 4 days, with the medium changed every 2 days. After the 4‐day culture, 2 µg mL^−1^ puromycin is added to the complete medium, and the cells are continuously cultured for an additional 4 days. After trypsinization with 0.25% trypsin‐EDTA, the cells are plated into a T25 flask. When the cells reach confluence, they undergo regular passaging and maintenance culture.

### Cells—Establishment of Cell High‐Fat Model

The F1 generation mouse USMCs were adjusted to a concentration of 2×10^9^ cells L^−1^. Control and high‐fat models of late pregnant mouse USMCs were obtained by incubating the cells with DMEM containing 200µM BSA and 200µM PA at 37 °C in a 5% CO_2_ incubator for 24 h.

### Cells—Cell Collagen Contraction Assay

First, cells were digested with trypsin, centrifuged, and resuspended in DMEM. Cell counting was performed using trypan blue staining. The cell density was adjusted to 12 00 000 cells/100 µL. Then, the collagen protein solution was prepared according to the instructions of the Cell Contraction Assay Kit (Cell Biolabs, America). Transfected cells were mixed with collagen solution at a ratio of 1:4, and 500 µL of the cell‐collagen mixture was added to a 24‐well plate and incubated at 37 °C in a cell culture incubator for 1 h until gelation occurred. DMEM containing 10% FBS was added to the solidified gel for further incubation. Gel images were captured at 0 and 12 h, and the area was measured using Image J software.

### Cells—Transfection of FABP4 siRNA into Cells

Mouse USMCs were transfected with FABP4 siRNA and negative control (NC) siRNA using jetPRIME transfection reagent (Polyplus, France) to achieve a transfection efficiency of 70–80%. The inhibitory effect of siRNA on FABP4 expression was detected by Western blotting and RT‐qPCR.

### Cells—Transfection of Mfn2 and CPT1A Overexpression Plasmid into Cells

The Mfn2 and CPT1A overexpression plasmid was designed by Shanghai GenePharma Co., Ltd. The Mfn2 plasmid vector was pcDNA3.1‐GP, with the following elements in order: CMV‐MCS‐SV40‐Neomycin. The length of the Mfn2 and CPT1A overexpression plasmid were respectively 5400 and 2322 bp, with cloning sites NotI/BamHI, bacterial resistance Ampicillin, and eukaryotic resistance Neomycin. Mouse USMCs were transfected with the Mfn2 and CPT1A overexpression plasmid, and negative control (PcDNA3.1) using jetPRIME transfection reagent (Polyplus, France) to achieve a transfection efficiency of 70–80%. The enhancement of Mfn2 and CPT1A expression by the overexpression plasmid was detected by Western blotting.

### 4D‐FastDIA Quantitative Proteomics—Protein Extraction

Samples were taken out from −80 °C and a suitable amount was weighed and transferred to a liquid nitrogen‐precooled mortar, where they were thoroughly ground into a powder with liquid nitrogen. Each group of samples was mixed with a lysis buffer at a volume ratio of 1:4 (8 m urea, 1% protease inhibitor, 3 µm TSA, 50 mM NAM) and subjected to ultrasonic lysis. The samples were centrifuged at 12 000 g for 10 min at 4 °C to remove cell debris. The supernatant was transferred to a new centrifuge tube, and protein concentration was measured using a BCA assay kit.

### 4D‐FastDIA Quantitative Proteomics—Trypsin Digestion

Equal amounts of protein from each sample were adjusted to the same volume using the lysis buffer for digestion. A final concentration of 20% TCA was slowly added, mixed by vortexing, and allowed to precipitate at 4 °C for 2 h. The samples were centrifuged at 4500 g for 5 min to discard the supernatant, and the precipitate was washed 2–3 times with precooled acetone. After drying, 200 mm TEAB was added to the precipitate, which was then sonicated to disperse it. Trypsin was added at a ratio of 1:50 (protease: protein, w/w) for overnight digestion. Dithiothreitol was added to a final concentration of 5 mm, and reduction was performed at 56 °C for 30 min. Subsequently, iodoacetamide was added to a final concentration of 11 mm, and the mixture was incubated in the dark at room temperature for 15 min.

### 4D‐FastDIA Quantitative Proteomics—Liquid Chromatography‐Mass Spectrometry Analysis

The peptides were dissolved in solvent A for liquid chromatography and separated using a NanoElute ultra‐high‐performance liquid chromatography system. Solvent A consisted of an aqueous solution containing 0.1% formic acid and 2% acetonitrile; solvent B was an acetonitrile‐water solution containing 0.1% formic acid. The liquid chromatography gradient was set as follows: 0–14 min, 6–24% B; 14–16 min, 24–35% B; 16–18 min, 35–80% B; and 18–20 min, 80% B, with a flow rate maintained at 500 nL min^−1^. After separation by the ultra‐high‐performance liquid chromatography system, the peptides were introduced into the capillary ion source for ionization before being analyzed by the timsTOF Pro mass spectrometer for data collection. The ion source voltage was set to 1.75 kV, and both the precursor ions and their secondary fragments were detected and analyzed using TOF. The data acquisition mode used was data‐independent parallel accumulation serial fragmentation (dia‐PASEF) mode, with the primary mass spectrometry scan range set at 300–1500 m/z (MS/MS scan range). After one primary mass spectrum was collected, 20 PASEF scans were conducted (MS/MS mode), and the secondary mass spectrum was scanned in the range of 400–850 m/z, with a window of 7 m/z.

### Western Blotting

Mouse muscle layer proteins were extracted using RIPA lysis buffer, phenylmethylsulfonyl fluoride (PMSF), and phosphatase inhibitors at a ratio of 100:1:2 (Beyotime Biotechnology, China). The total protein concentration was determined using the BCA protein assay kit (Beyotime Biotechnology, China). Protein samples were separated by SDS‐PAGE and transferred onto polyvinylidene fluoride (PVDF) membranes (Millipore, USA). After blocking with 5% skim milk in Tris‐buffered saline (TBS)‐Tween at room temperature for 1 h, the membranes were incubated with primary antibodies against FABP4 (rabbit monoclonal antibody, 1:2000 dilution, Abcam Cat# ab76659, RRID:AB1523585), AKT (rabbit monoclonal antibody, 1:1000 dilution, Abcam Cat# ab59285, RRID:AB940187), AKT S473 (rabbit monoclonal antibody, 1:1000 dilution, Abcam Cat# ab8932, RRID:AB306867), Mfn2 (mouse monoclonal antibody, 1:1000 dilution, Abcam Cat# ab56889, RRID:AB2142629), CPT1A (rabbit, monoclonal antibody, 1:500 dilution, Affinity Biosciences Cat# DF12004, RRID:AB_2844809), PPAR γ (rabbit, monoclonal antibody, 1:1000 dilution, Affinity Biosciences Cat# AF6284, RRID:AB_2835135), PGC‐1 alpha (rabbit, monoclonal antibody, 1:1000 dilution, Abcam Cat# ab191838, RRID:AB_2721267), LDHA (rabbit, monoclonal antibody, 1:1000 dilution, Affinity Biosciences Cat# DF6280, RRID:AB_2838246), GLUT1 (rabbit, monoclonal antibody, 1:1000 dilution, Abcam Cat# ab115730, RRID:AB_10903230), GLUT4 (rabbit, monoclonal antibody, 1:1000 dilution, Abcam Cat# ab188317, RRID:AB_2890624), PDK1 (rabbit, monoclonal antibody, 1:2000 dilution, Abcam Cat# ab207450, RRID:AB_3064841), and HIF‐1 alpha (rabbit, monoclonal antibody, 1:1000 dilution, Abcam Cat# ab179483, RRID:AB_2732807) overnight at 4 °C. After washing with TBS‐Tween three times for 10 min each, the membranes were incubated with horseradish peroxidase‐conjugated secondary antibodies (goat anti‐mouse or goat anti‐rabbit, 1:10 000 dilution, Elabscience, China) at room temperature for 2 h. Chemiluminescent detection was performed to visualize the immunoblots. Protein expression was quantitatively analyzed using ImageJ software.

### Real‐Time Quantitative Polymerase Chain Reaction (RT‐qPCR)

Total RNA was extracted from muscle tissue using TRIzol, chloroform, and isopropanol (Sigma Aldrich, USA) according to the kit instructions. The concentration and purity of the extracted RNA were measured using a NanoDrop 2000 spectrophotometer (Thermo Scientific, USA). One microgram of RNA was reverse transcribed into cDNA using the PrimeScript RT reagent Kit with gDNA Eraser (Takara BIO, Japan). Primers were designed and synthesized by Sangon Biotech (China). The primer sequences for FABP4 and β‐actin were as follows: FABP4 forward 5′‐GGAAGCTTGTCTCCAGTGAAAAC‐3′, reverse 5′‐TGACCAAATCCCCATTTACGC ‐3′; β‐actin forward 5′‐GGCTGTA TTCCCCTCCA tcg ‐3′, and reverse 5′‐CCAGTTGGTAACAA TGCCA TGT‐3′. The RT‐qPCR reaction mixture consisted of cDNA, a pair of primers, double‐distilled water, and SYBR Green Master Mix (Roche, Switzerland). The amplification reaction was performed using a LightCycler 480 instrument (Roche, Switzerland). The expression level of the target gene was quantified using the arithmetic formula 2^−ΔΔCt^, with β‐actin as the reference gene for normalization.

### Immunostaining and Confocal Microscopy—Lipid Detection in USMCs

The cells were fixed with 4% paraformaldehyde, gently washed with PBS for 15 min at room temperature, and then incubated with Nile Red (1:1000, 72 485, Sigma, St. Louis, USA) in PBS for 30 min in the dark. After shaking in PBS for 30 min, confocal images were obtained using a Zeiss LSM 800 confocal microscope with a 63X oil objective lens.

### Immunostaining and Confocal Microscopy—FAO Activity Detection

The cultured USMCs were washed with fresh HEPES‐buffered saline (HBS) twice. Then FAO Blue dye was added into the cells to make the working concentration up to 10 µm, and the cells were incubated for 30 min. Finally, the cells were washed with HBS again, and observed under live conditions with blue fluorescence (Ex. 405 nm/ Em. 430–480 nm).

### Immunostaining and Confocal Microscopy—Rhod‐2 AM Mitochondrial Calcium Detection

Rhod‐2 AM was diluted in calcium‐magnesium‐containing Hanks' solution at a ratio of 1:1000 to prepare a working solution with a final concentration of 4 µm. A certain amount of NaBH4 was added and thoroughly mixed until colorless, followed by the addition of Pluronic F‐127 to a final concentration of 0.003%. After washing the cells with PBS once, the working solution was added and incubated at 37 °C in a dark incubator for 40 min. After washing with PBS once, calcium‐magnesium‐free Hanks' solution was added and incubated at 37 °C in a dark incubator for 30 min to fully de‐esterify. After washing with PBS twice, the mitochondrial green fluorescent probe was diluted in calcium‐magnesium‐free Hanks' solution at a ratio of 1:5000 and added to each group, followed by incubation at 37 °C in a dark incubator for 15 min. After washing with PBS twice, the slides were placed in a laser confocal microscope, and the fluorescence intensity of Rhod‐2 AM was detected with an excitation wavelength of 543 nm and an emission wavelength of 560–660 nm, while the mitochondrial marker was detected with an excitation wavelength of 488 nm and an emission wavelength of 505–525 nm.

### Immunostaining and Confocal Microscopy—Mitochondrial Membrane Potential Detection in USMCs

One milliliter of JC‐1 staining working solution was added and thoroughly mixed. The cells were incubated at 37 °C in a cell culture incubator for 20 min. After incubation, the supernatant was removed, and the cells were washed twice with JC‐1 staining buffer. 2 mL of cell culture medium, which can contain serum and phenol red, was added. Confocal images were obtained using a Zeiss LSM 800 confocal microscope with a 63X oil objective lens. When detecting JC‐1 monomers, the excitation light can be set at 490 nm and the emission light at 530 nm; when detecting JC‐1 aggregates, the excitation light can be set at 525 nm and the emission light at 590nm.

*For subcellular localization of IP3R1, GRP75, and VDAC1 proteins in cells*, the cells were fixed with 4% paraformaldehyde, permeabilized with 0.05% Triton X‐100, and blocked with 3% bovine serum albumin at 37 °C for 2 h. The cells were then incubated with primary antibodies against IP3R1 (rabbit monoclonal antibody, 1:200 dilution, ABclonal Cat# A4436, RRID:AB2863274), GRP75 (mouse monoclonal antibody, 1:100 dilution, Huabio Cat# EM1701‐23, RRID:AB3068751), VDAC1 (rabbit monoclonal antibody, 1:200 dilution, ProSci Cat# 28–243, RRID:AB10909264), and VDAC1 (mouse monoclonal antibody, 1:200 dilution, Creative Biomart Cat# CABT‐38446MH, RRID:AB11471152) overnight at −4 °C in 3% BSA. The samples were washed with PBS, and DAPI staining was performed for 30 min. Confocal images were obtained using a Zeiss LSM 800 confocal microscope with a 20X objective lens.

### Immunostaining and Confocal Microscopy—Detection of Mitochondria‐Associated Protein and Fatty Acid Oxidation Essential Protein in USMCs

After the procedures of fixation, permeability and blocking, the cells were incubated with primary antibodies against CPT1A (rabbit, monoclonal antibody, 1:200 dilution, Affinity Biosciences Cat# DF12004, RRID:AB_2844809), PPAR γ (rabbit, monoclonal antibody, 1:200 dilution, Affinity Biosciences Cat# AF6284, RRID:AB_2835135), PGC‐1 alpha (rabbit, monoclonal antibody, 1:200 dilution, Abcam Cat# ab191838, RRID:AB_2721267), and TOMM20 (rabbit, monoclonal antibody, 1:250 dilution, Abcam Cat# ab186735, RRID:AB_2889972) overnight at 4 °C. After the cells were washed three times with PBS, they were incubated with the secondary antibody conjugated with Alexa Fluor 488 goat anti‐rabbit IgG (green) for 2 h at 37 °C. Then cells were washed and stained with Hoechst for 10 min and photographed.

### Mitochondrial Respiration Assay

4000 cells were seeded in a Seahorse detection 96‐well plate. After the cells adhered to the plate, the medium was changed the next day and incubated at 37 °C with 5% CO_2_. On the day before measuring the OCR, 200 µL of hydration solution was added to each well and incubated overnight at 37 °C without CO_2_. On the measurement day, XF‐96 assay medium was prepared by adding 500 µL of glutamine (100×), 500 µL of sodium pyruvate solution, and 200 µL of 45% D‐(+) glucose solution to XF‐96 base medium to a final volume of 50 mL. The pH was adjusted to 7.4 with KOH. Oligomycin, an inhibitor of mitochondrial oxidative phosphorylation, FCCP, and rotenone/antimycin A (Rot/AA) were diluted in XF‐96 assay medium to final concentrations of 2.4, 1, and 0.6 µm, respectively. After washing the cells twice with XF‐96 assay medium, 180 µL of XF‐96 assay medium was added to each well and incubated at 37 °C without CO_2_ for 1 h. Oligomycin, FCCP, and Rot/AA were sequentially added in 20, 22, and 25 µL, respectively, to the drug plate. After aspirating the drugs, the cell plate was added, and the OCR was measured.

### ATP Measurement

According to the manufacturer's protocol (Beyotime Biotechnology), cells were digested, counted and centrifuged, and 200 µL lysate was added. For tissue, 20 mg fresh myometrium tissue was added to 200 µL lysate, then ground, and the protein content was determined through BCA method. The lysate was collected and centrifuged at 4 °C for 5 min to obtain the supernatant. The detecting solution was added to a 96‐well plate and incubated at room temperature for 3 min. The supernatants were then added to the wells and measured with a luminometer after the mixture. The corresponding luminescence was calculated according to the standard curve. Depending on the cell number or the protein concentration of the tissue, the ATP level was calculated, and data were normalized to fold change.

### Glucose Uptake Measurement

Cells were seeded in a 96‐well plate at a density of 5000 cells per 100µl of DMEM with 10% fetal bovine serum. Each well was added with 50µl of prepared 1 mm 2DG, briefly shaken, and incubated at room temperature for 10 min. On the day before measurement, the culture medium was removed and replaced with 100 µL of serum‐free DMEM. On the measurement day, 100 µL of serum‐ and glucose‐free DMEM ± 1 µm insulin was added to each well, and incubated at 37 °C with 5% CO_2_ for 1 h. The culture medium was removed, and 50 µL of 0.1 mm 2DG dissolved in PBS was added. The plate was incubated at 25 °C for 30 min. Then, 25 µL of stop buffer was added and the samples were briefly shaken. After that, 25 µL of neutralization buffer was added and the samples were briefly shaken. Finally, 100 µL of 2DG6P detection reagent was added and the samples were briefly shaken. The plate was incubated at 25 °C for 1 h. Luminescent signals were recorded using a multifunctional microplate reader with a signal integration time of 0.3–1 s.

### Lactic Acid Measure

According to the manufacturer's protocol (Nanjing Jiancheng Bioengineering Institute), the fresh myometrium tissue was washed, and ground with cold normal saline, and the BCA method was used to determine the protein content. Then, the supernatant was collected after centrifugation, and mixed with the constituents in the lactic acid assay kit. The sample was heated at 37 °C for 10 min, and measured for absorbance at 530 nm wavelength. Data were presented as the lactic acid concentration (mm/g), and normalized to fold change. As the lactic acid in cells, the collected cell culture supernatants were used to measure the lactic acid level according to the manufacturer's instructions. Data was presented as the lactic acid concentration (mm/cells), and normalized to fold change.

### In Situ Proximity Ligation Assay (PLA)

The Duolink II PLA (Olink Bioscience) allows for the evaluation of the proximity and potential interaction of two proteins as individual fluorescent dots (<40 nm) under a microscope. The cell fixation and permeabilization methods are similar to immunofluorescence experiments. Subsequent blocking, antibody hybridization, proximity ligation, and detection steps were performed according to the manufacturer's recommendations. Confocal images were obtained using a Zeiss LSM 800 confocal microscope with a 10X objective lens. The signals were quantified using BlobFinder software and expressed as the percentage of spots per cell nucleus compared to the control group.

### Data Analysis

Data analysis was performed using GraphPad Prism 8.0 software. All data are presented as mean ± standard deviation (SD). Unpaired Student's *t*‐test was used for comparisons between the two groups. Two‐tailed Student's *t*‐test was used for PLA fluorescence signal analysis. A *p*‐value of 0.05 was considered statistically significant.

### Ethics for Animal Experiments and Human Samples

Experiments with mice were conducted according to institutional guidelines for animal ethics and approved by the Anhui Medical University Experimental Animal Welfare and Ethics Committee (No. LLSC20211056, No. LLSC20232218). Experiments with human samples followed the protocol reviewed and approved by the First Affiliated Hospital of Anhui Medical University Ethics Committee for the Protection of Human Subjects in Research and Tissue Collection (PJ2023‐01‐60). The patients/participants provided their written informed consent to participate in this study.

## Conflict of Interest

The authors declare no conflict of interest.

## Author Contributions

X.L., H.Y., and R.T. contributed equally to this work as co‐first authors. Y.Z., C.Y. and L.X. conceived and designed the experiments. L.X., Y.H. and T.R. conducted the experiments and drafted the first draft. L.X., Y.H., T.R., W.X., X.T., X.C., L.T., D.X., C.Q. and Y.B. contributed to the data and the discussion. L.X., Y.H. and T.R. contributed equally to this study as first authors. All authors read and approved the final paper.

## Supporting information



Supporting Information

## Data Availability

The data that support the findings of this study are available from the corresponding author upon reasonable request.
