# Supplementary material for: FABP4 as a Mediator of Lipid Metabolism and Pregnant Uterine Dysfunction in Obesity
Source: Adv Sci (Weinh). 2025 Apr 7;12(25):2501077. doi: 10.1002/advs.202501077 (PMC12225006; doi:10.1002/advs.202501077)
Supplement: Supplementary file 1 — Supporting Information [file ADVS-12-2501077-s001.pdf]

ADVANCED SCIENCE
Open Access

Supporting Information

for *Adv. Sci.*, DOI 10.1002/advs.202501077

FABP4 as a Mediator of Lipid Metabolism and Pregnant Uterine Dysfunction in Obesity

Xuan Li, Huihui Yu, Ruixian Tian, Xingxing Wang, Ting Xing, Chenyi Xu, Tengteng Li, Xue Du, Qianqian Cui, Biao Yu, Yunxia Cao and Zongzhi Yin**

Supplemental Figures

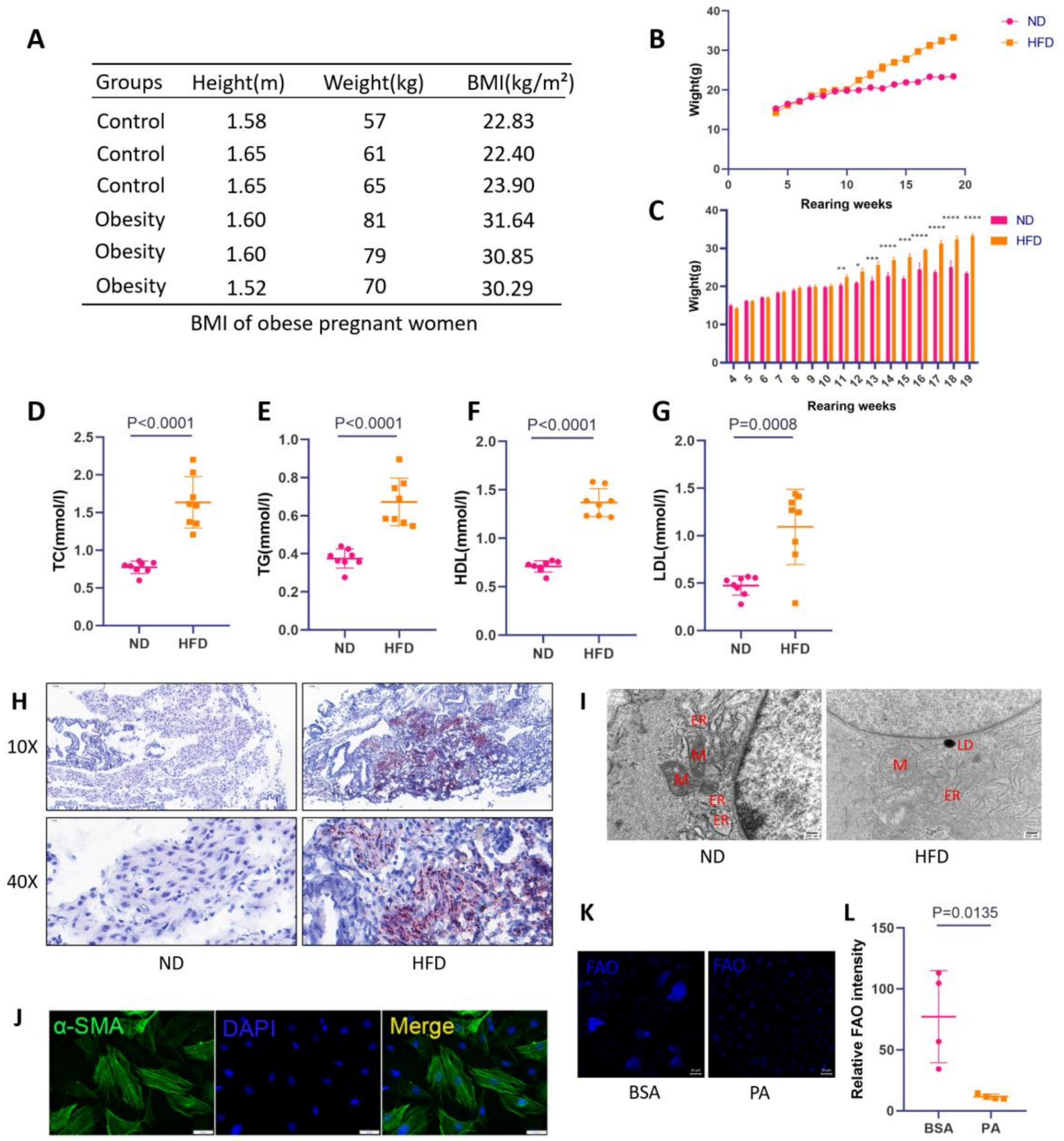

Fig.S1 Obese pregnant women and HFD mice were modeled.

A, BMI of obese pregnant women (n = 3 people per group). **B, C**, Changes in body weight after 16 weeks of ND or HFD feeding (n = 5 mice per group). **D-G**, Serum TG, Serum TC, Serum LDL, Serum HDL (n = 8 samples per group, pooled from 3 mice per sample). **H**, Oil Red O staining of uterine tissue sections shows increased lipid deposition in the uterine smooth muscle tissue of HFD obese mice compared to the ND group (n = 2 mice per group) , scale bar=20 μm or 100 μm. **I**, Representative images showing mitochondria (M), ER, and LDs. **J**, Representative images of a-SMA (green) staining for USMCs and DAPI (blue) labeling for nucleus (n = 3 samples per group), scale bar=10 μm. **K**, Representative photomicrographs of FAO staining (n = 4 samples

per group), scale bar=10 μm. All experiments were repeated 2-3 times with consistent results. The values are expressed as the means ± SDs. *$p<0.05$, **$p<0.01$ and ***$p<0.001$ versus the control group.

A

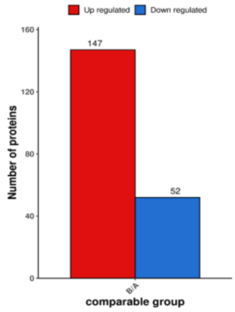

B

Proteomics FABP4 protein assay

Groups	Detected values			Ratio	P value
HFD	1.120	1.569	1.449	2.222	0.002398657
ND	0.648	0.660	0.554		

C

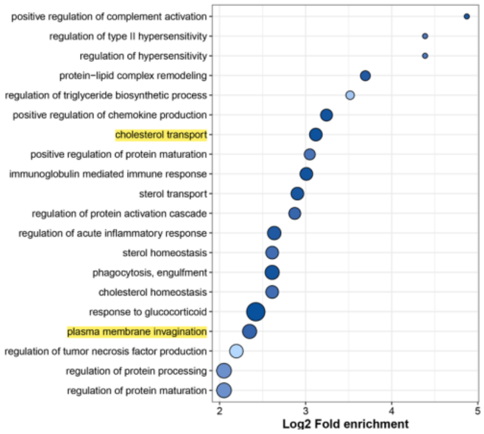

D

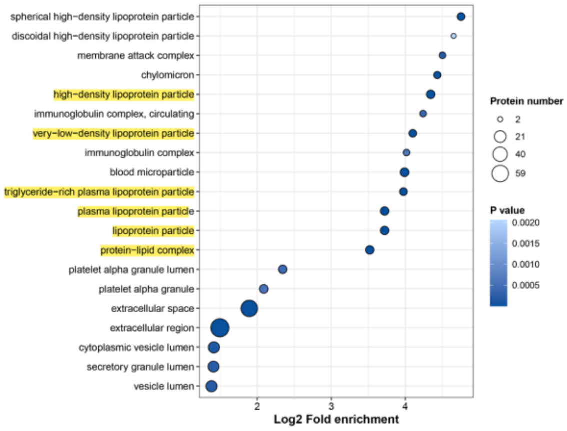

E

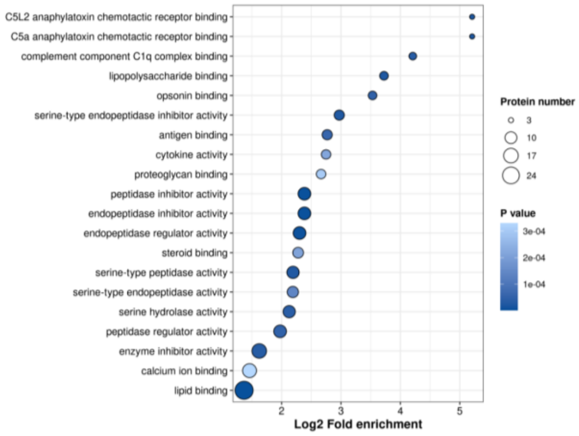

F

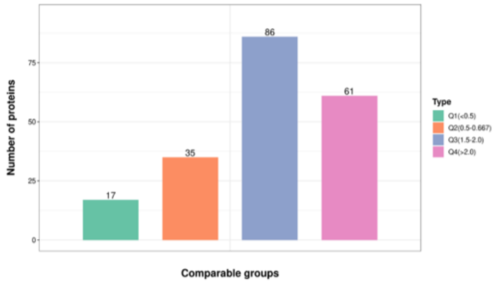

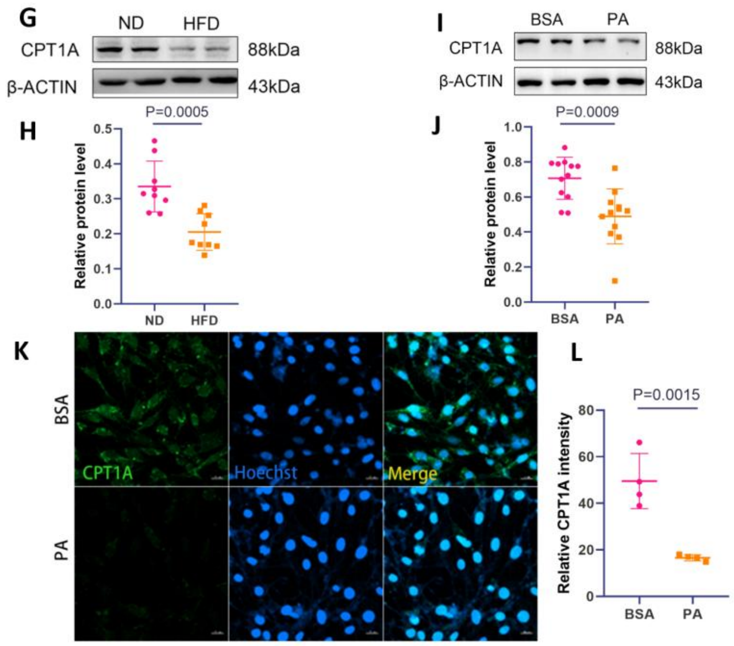

Fig.S2 The 4D-FastDIA quantitative proteomic analysis

A, Basic statistical graph of mass spectrometry data results. **B**, 4D-FastDIA quantitative proteomic analysis of FABP4 protein. **C**, GO enrichment analysis on the differentially expressed proteins between obesity group and control group. Key factors analyzed included p-value, protein number, and degree of enrichment. The results showed that the differentially expressed proteins were mainly involved in cholesterol transport and endocytosis**. D**, Cellular component analysis indicated that the differentially expressed proteins were mainly related to lipoprotein particles and complexes. **E**, The molecular function analysis showed that the differentially expressed proteins were mainly involved in functions which included lipoprotein binding and calcium ion binding. **F**, To compare the similarities and differences in functions among proteins with different fold changes, we divided the proteins into four groups (Q1-Q4) based on the fold change. **G, H**, Representative Western blots and quantitative analysis of CPT1A expression in uterine smooth muscle tissue of HFD-fed pregnant mice (n = 9 samples per group, pooled from 3 mice per sample). **I, J**, Representative Western blots and quantitative analysis of CPT1A expression in USMCs of the high-fat model (n = 12 samples per group). **K, L**, Immunofluorescent image of myometrium with BSA and PA. CPT1A was stained in green, nuclei were stained in blue (n = 4 samples per group, pooled from 3 mice per sample), scale bar=10 μm. All experiments were repeated 2-3

times with consistent results. The values are expressed as the means ± SDs. $^{*}p<0.05$, $^{**}p<0.01$ and $^{***}p<0.001$ versus the control group.

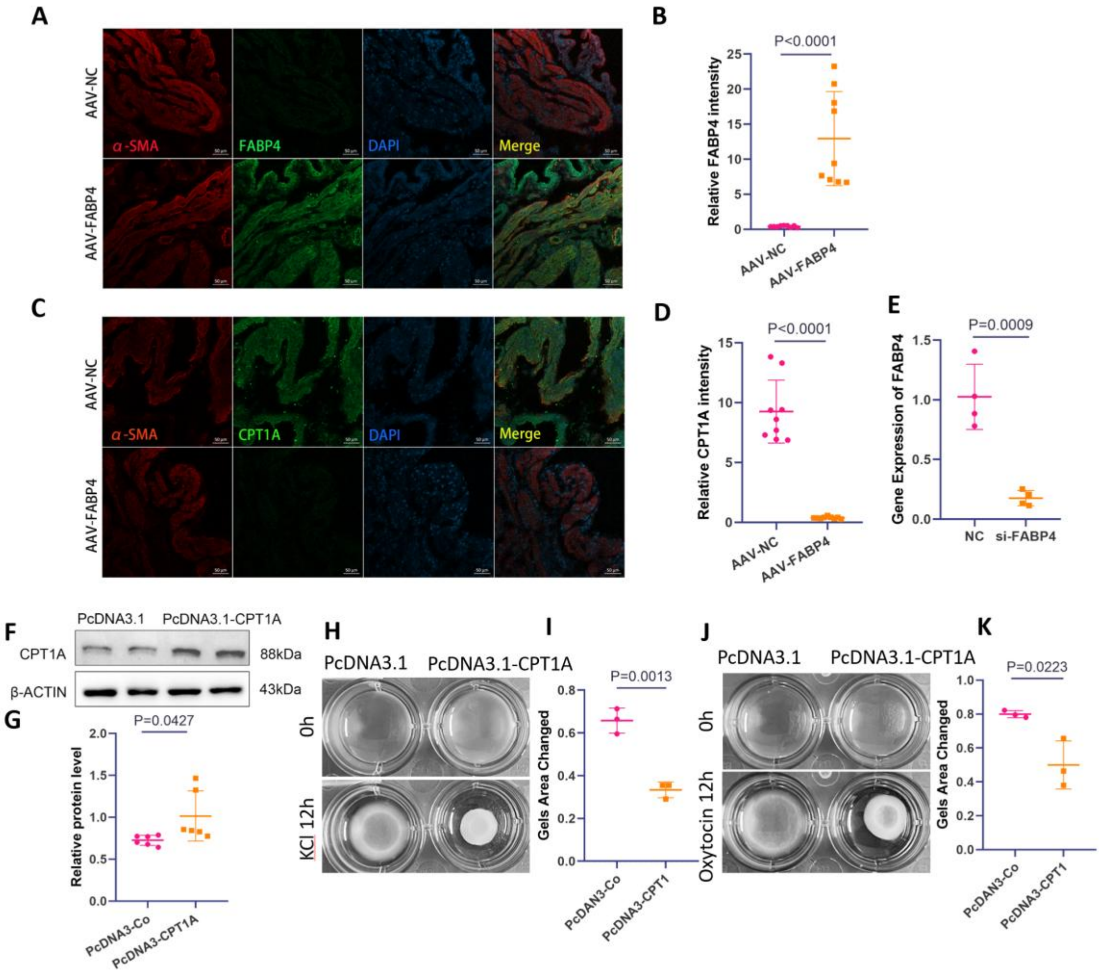

Fig.S3 FABP4 promotes lipid accumulation and disrupts uterine contractions.

A-**D**, Immunofluorescent image of myometrium from mice in AAV-NC group and AAV-FABP4 group. α-SMA was stained in red, FABP4 protein and CPT1A protein were stained in green, nuclei were stained in blue (n = 9 samples per group, pooled from 3 mice per sample), scale bar = 50 μm. **E**, RT-qPCR analysis to assess the efficiency of FABP4 siRNA transfection (n = 4 samples per group, pooled from 3 mice per sample). **F, G**, Effect of CPT1A overexpression plasmid transfection for 48 hours (n = 6 samples per group, pooled from 3 mice per sample). **H, I**, Collagen contraction images and statistical results after KCl stimulation in USMCs transfected with CPT1A overexpression and treated with PA (n = 3 mice per group). **J, K**, Collagen contraction images and statistical results after oxytocin stimulation in USMCs transfected with CPT1A overexpression and treated with PA (n = 3 mice per group). All experiments were repeated 2-3 times with consistent results. The values are expressed as the means ± SDs. *p<0.05, **p<0.01 and ***p<0.001 versus the control group.

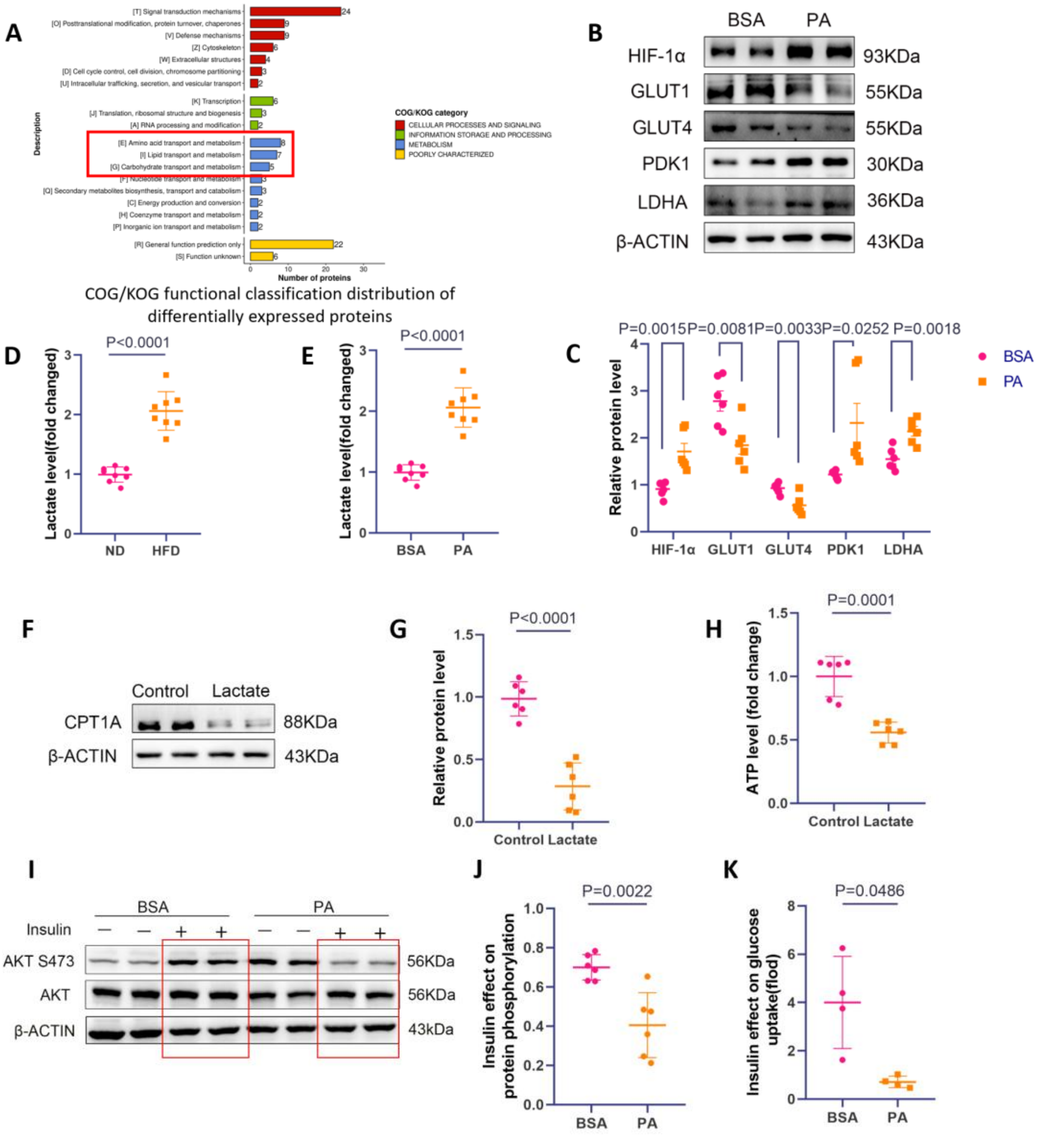

Fig.S4 High lipid damages glucose metabolism, followed lactic acid accumulation and insulin resistance.

A, 4D-FastDIA quantitative proteomic analysis revealed that the differentially expressed proteins in uterine muscle tissue of both model groups are involved in glucose transport and metabolism. The x-axis represents the number of differentially expressed proteins corresponding to each entry, and the y-axis represents the COG/KOG category number and description. The color represents the category of each COG/KOG entry (n = 3 mice per group). **B, C**, Representative Western blots and quantitative analysis of HIF-1α, GLUT1, GLUT4, PDK1 and LDHA expression in PA-treated cells (n = 6 samples per group, pooled from 3 mice per sample). **D**, The serum

and tissue lactate levels were measured using the Lactate Assay Kit (n = 8 samples per group, pooled from 3 mice per sample). **E**, The lactate levels in PA-treated cells culture supernatant were measured with the Lactate Assay Kit (n = 9 for BSA group; n = 8 for FABP4 PA group, pooled from 3 mice per sample). **F, G**, Representative Western blots and quantitative analysis of CPT1A expression in lactate-treated cells (n = 6 samples per group, pooled from 3 mice per sample). **H**, The ATP levels in the uterine myometrium of late-pregnant mice under lactic acid incubation were measured using the ATP Assay Kit (n = 3 mice per group). **I, J**, Changes in phosphorylation of AKT Ser473 and total Akt expression levels, ratio of phosphorylated AKT Ser473 to total AKT (n = 6 samples per group, pooled from 3 mice per sample). **K**, To investigate the effect of high-fat environment on glucose metabolism in myometrium of pregnant mice, the Glucose Uptake-GloTM Assay was performed on PA-treated cells, revealing a weakened glucose uptake ability (n = 4 samples per group, pooled from 3 mice per sample). All experiments were repeated 2-3 times with consistent results. The values are expressed as the means ± SDs. *$p<0.05$, **$p<0.01$ and ***$p<0.001$ versus the control group.

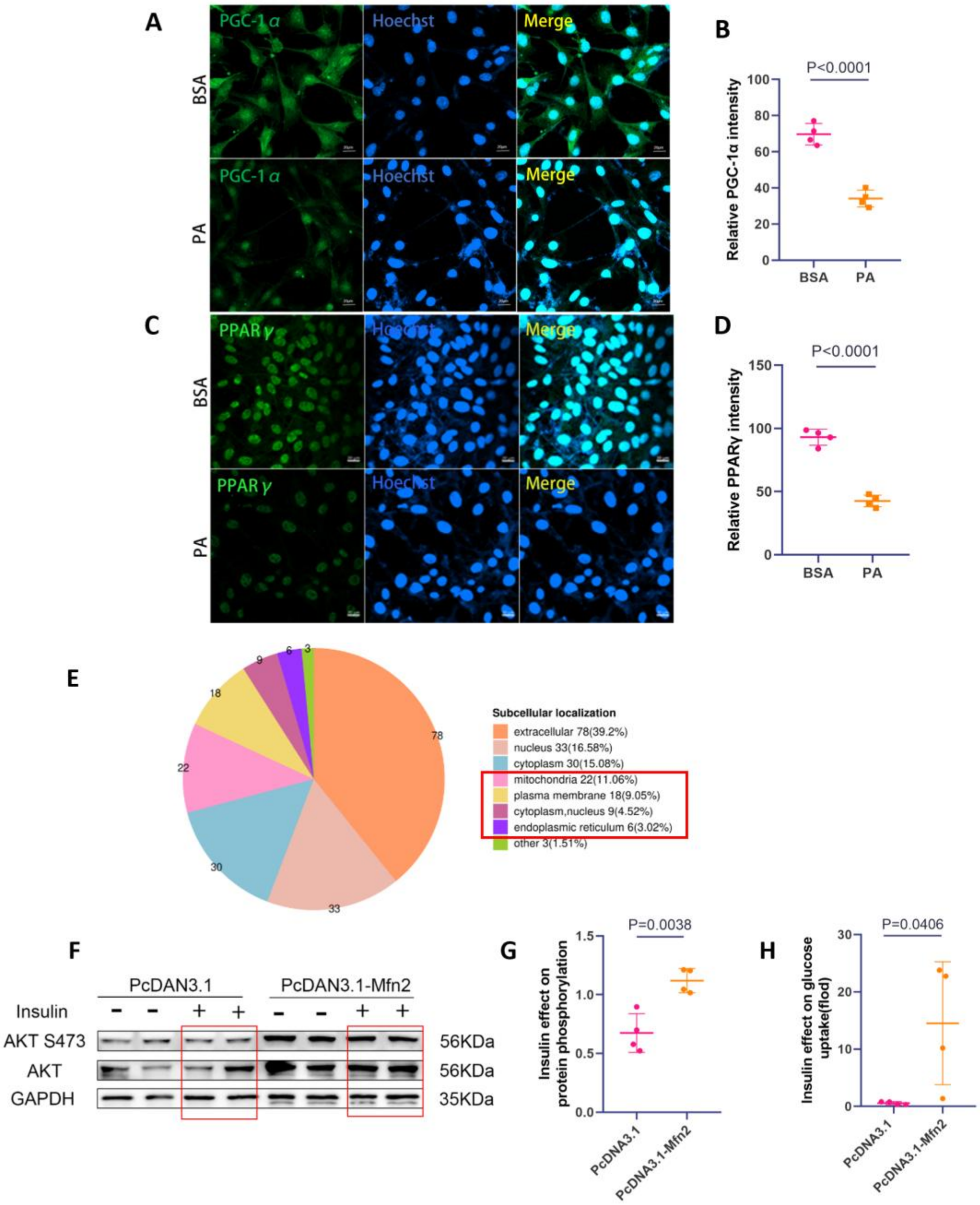

Fig.S5 Levels of insulin resistance following Mfn2 overexpression.

A-**D**, Immunofluorescent image of myometrium with BSA and PA. PGC-1αand PPAR γ were stained in green, nuclei were stained in blue (n = 4 samples per group, pooled from 2 mice per sample), scale bar =10μm. **E**, Subcellular localization distribution of differentially expressed proteins. **F, G**, Representative Western blots and quantitative analysis of AKT phosphorylation changes in USMCs transfected with Mfn2 overexpression plasmid and treated with PA after insulin stimulation (n = 4 samples per group, pooled from 2 mice per sample). **H**, Glucose Uptake-GloTM Assay results showing enhanced glucose uptake in late-pregnancy mouse USMCs transfected with

Mfn2 overexpression plasmid and treated with PA (n = 4 samples per group, pooled from 3 mice per sample). All experiments were repeated 2-3 times with consistent results. The values are expressed as the means ± SDs. *p<0.05, **p<0.01 and ***p<0.001 versus the control group.
